# Spatial distribution of benthic foraminifera in the Lagos Lagoon (Nigeria): Tracing the impact of environmental perturbations

**DOI:** 10.1371/journal.pone.0243481

**Published:** 2020-12-07

**Authors:** Olugbenga T. Fajemila, Nisan Sariaslan, Martin R. Langer

**Affiliations:** 1 Department of Geological Sciences, Osun State University, Osogbo, Nigeria; 2 Institut für Geowissenschaften, Paläontologie, Rheinische Friedrich-Wilhelms-Universität, Bonn, Germany; CIIMAR Interdisciplinary Centre of Marine and Environmental Research of the University of Porto, PORTUGAL

## Abstract

Lagos Lagoon is among Africa’s largest estuarine ecosystems, bordered by one of the fastest growing megacities in the world and the ultimate repository of contaminants carried in industrial, municipal and agricultural wastes. The high levels of pollutants have progressively deteriorated the water quality, adversely affected lagoon ecosystems, impacted the livelihood of the coastal population and pose serious risks to human health. Benthic foraminifera are excellent proxies and sensitive bioindicators of environmental disturbances but comprehensive studies on the structure, distribution, diversity and impact of pollution upon foraminiferal communities have not yet been conducted in the Lagos Lagoon. To demonstrate the potential of foraminifera as proxies of environmental perturbations, benthic foraminifera were investigated on a lagoon-wide basis. Lagos Lagoon comprises areas that range from low levels of direct impact to those of severely affected by various forms of anthropogenic disturbance. The goals of this study are to analyze patterns of distribution and species richness, to document foraminiferal community structures, and to identify taxa that track documented records of pollution in Lagos Lagoon sediments. Heat maps were generated from abundance records for selected species to illustrate environmental preferences and relative resistance levels to individual forms of anthropogenic disturbance. Sediments were analyzed for a range of physicochemical properties, via a multi-parameter sensor probe-device, including temperature, pH, depth and total dissolved solids (TDS). Quantitative analysis of 24 sediment samples yielded a total 3872 individuals of benthic foraminifera that belong to 42 species and 25 genera. They comprise 10 porcellaneous, 22 hyaline perforate and 10 agglutinated species. *Ammobaculites exiguus*, *Ammotium salsum*, *Ammonia aoteana*, *Ammonia convexa* and *Trochammina* sp. 1 have been found to be the most abundant species. For the first time, the complete present-day foraminifera fauna is illustrated here via scanning electron microscopy. The features recorded allow to assess the spatial effects of pollution upon foraminiferal assemblages on a lagoon-wide basis. The data generated may ultimately form the basis to assess the progressive deterioration of Lagos Lagoon ecosystems from cores by using benthic foraminifera as bioindicators of environmental perturbation.

## Introduction

**Fig 1:**
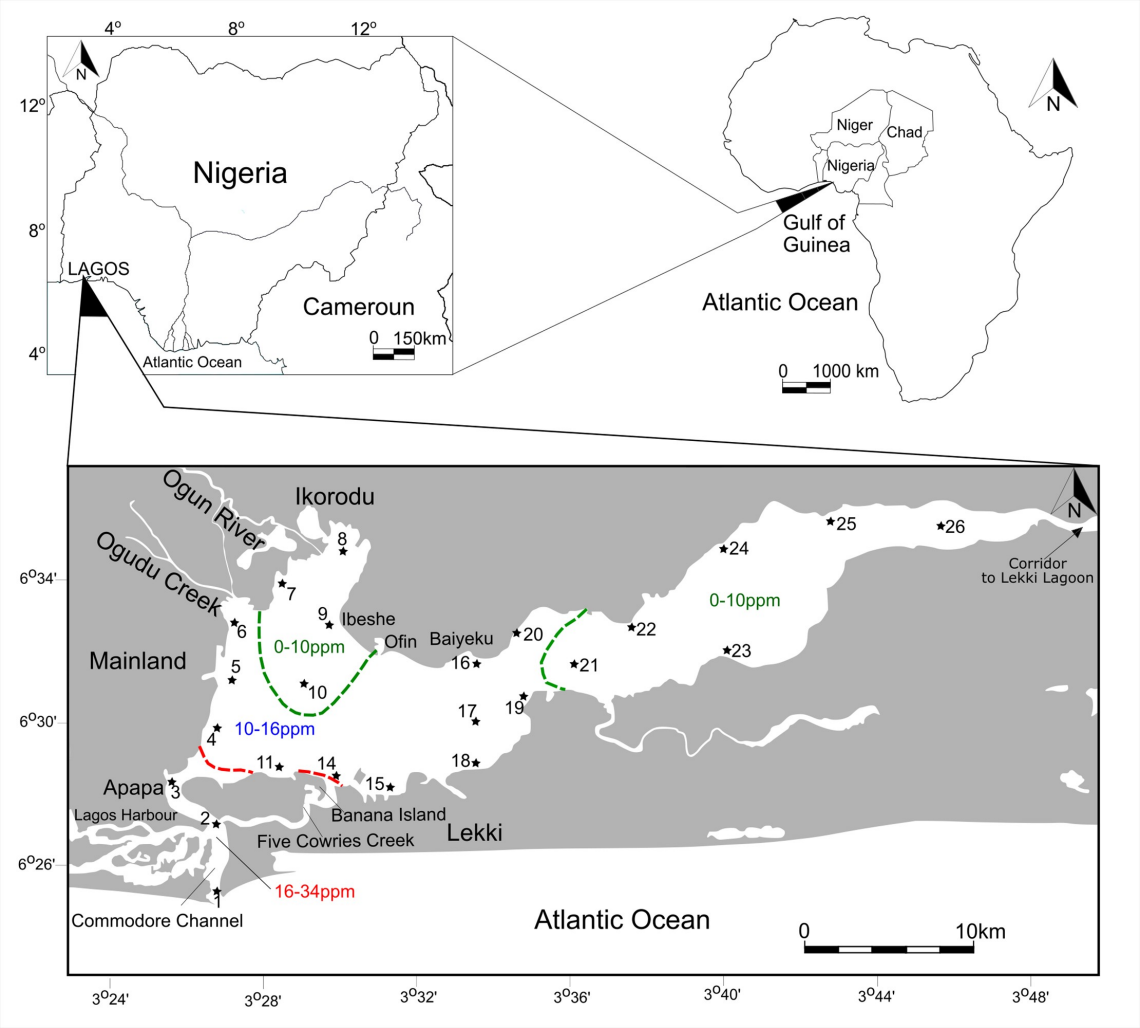
Location of the sample sites and generalized salinity contours in Lagos Lagoon, Gulf of Guinea (Nigeria). Salinity in Lagos Lagoon varies by season and location, with the freshest water being in the northern and the eastern portion of the lagoon. The salinity contours provided cover the range of seasonal variation [8, 10, 11]. Salt water entering the lagoon via the main Atlantic entrance channel and the Five Cowries Creek mingles with lagoon water and creates a brackish water environment with decreasing values towards the distal ends of the lagoon. The Osun River discharges its waters into Lekki lagoon, a large expanse of shallow freshwater situated to the east of Lagos Lagoon, and connected to it via a narrow corridor.

Lagos Lagoon (Nigeria) is the largest lagoon system in the Gulf of Guinea with more than 6,000 km^2^ of surface area. The lagoon is situated between the Atlantic Ocean and Lagos, one of the fastest growing megacities in the world (Fig 1). The rapid population growth and industrial development, has made Lagos the economic hub and financial focal point of Nigeria with one of the largest seaports along the African coastline. Surrounded by a population of more than 20 million people, the lagoon has become the ultimate sink for the disposal of industrial, agricultural and domestic wastewaters. Lagos Lagoon ranks first among the most polluted African ecosystems (data from WHO and Africa UN Environment) and is primarily impacted by effluents from the oil and textile industry and urban sewage carried by the Ogun and Osun rivers. These led to high concentrations of heavy metals (e.g., copper, zinc, manganese, lead, iron, nickel) in the lagoon environment. Polycyclic aromatic hydrocarbons (e.g., naphthalene, phenanthrene, pyrene) have been found at considerable levels within the polluted western section of the lagoon (e.g., [1–4]). Excessive sand mining and dredging activities contribute more to the disruption of the ecosystem in the lagoon. Moreover, the lagoon is heavily exploited by fishing activities and aquaculture, leading to further environmental degradation and accompanying changes in water quality with biological consequences for biotas in the environment. With the expansion of Lagos City, large-scale destruction, deforestation and pollution of the mangroves forests have severely modified ecosystems along the western lagoon shores and resulted in the domination of tidal swamps by floating water-hyacinth (*Eichhornia crassipes*), saltgrasses and weeds [5]. These observations constitute the motivation for this study, which aims to investigate the structure, distribution and composition of foraminiferal assemblages in order to better understand the degree of environmental perturbation and to identify potential taxa as tracers of pollution. Previous studies on modern foraminifera from the Lagos Lagoon are limited, restricted to a few selected sites and mostly focused on the western part [6–8]. In a recent study, Philipps et al. [9] identified 20 species of benthic foraminifera from the western part of Lagos Lagoon and considered pollution as a driving force for harbor samples that are barren of foraminifera. Here we provide the first lagoon-wide analysis of present-day benthic foraminifera, illustrate the entire fauna, identify potential bioindicators and sites of pollution and highlight aspects to be considered in future in biomonitoring studies.

### Environmental setting

Lagos Lagoon is separated from the Atlantic Ocean by a long sandspit and drains its water via the comparatively narrow Commodore Channel into the Atlantic. The most densely populated areas including clusters of industry are spread along the lagoon's southwestern and western shorelines. Due to the limited exchange with marine waters, the Lagos Lagoon system experiences restricted marine and mainly low salinity, brackish and freshwater conditions [8, 10, 11]. Currents in Lagos Lagoon are strongly constrained by the tidal regime and freshwater discharge from Ogun River [10]. At high tide, incoming waters flow from the Atlantic Ocean into the harbor and the lagoon through the Commodore Channel and the Five Cowries Creek and are mainly directed towards the east. At low tide, the direction of the currents is reversed. Moreover, salinity varies substantially with the wet and dry seasons and is strongly impacted by the introduction of fresh water from rain, rivers and saline water from the ocean (Fig 1). Rivers Ogun and Osun empty into the lagoon through the northern and eastern corridors, reducing the salinity at these sectors tremendously and create fan-deltas. Minimal salinity values are recorded during the high rainfall months (July, August, September) and higher values are present during the dry season. In general, the western sector of the lagoon experiences higher salinity because of its interactions with the Atlantic Ocean. During the dry season, the influx of river water is low and salinities rise to about 30‰ around the entrance channel, to ~16‰ in the southwestern area, and to 8–10% in the central part. Towards the east and near the mouth of the Ogun River, salinities decrease further. The river input, however, is so large during the raining season, that the lagoon is fresh to brackish throughout and salinities in the central lagoon area drop to 3‰, to below 1‰ in the eastern sector and to 0‰ at the mouth of the Ogun River [11]. During the dry season, bottom water hypoxia events were recorded in the eastern sector of Lagos Lagoon [12].

The lagoon is known to have a wide range of sediments from mangrove swamp to muddy and sandy foreshores with either slight or pronounced wave action according to the degree of exposure, depth and the extent of the open water [9, 10]. Thus, a wide variety of mixed deposits containing different proportions of coarse sand, fine sand, silty mud, and mud cover the lagoon floor. Sediment samples were found to consist of dark grey, organic-rich muddy sand and fine- to coarse-grained sand containing various abundances of mollusk shells.

## Material and methods

Sampling was carried out between May 24–26, 2019, which corresponds to the beginning of the rainy season in Nigeria. The sampling was conducted in collaboration with the Nigerian Institute of Oceanography and Marine Research (NIOMR), as necessitated by the overall extent of the lagoon and the laborious logistics required (no specific permits were required for the described field studies). A total of twenty-four sites were sampled by boat with a Van Veen grab sampler and sediment was scraped off from the top 2 cm. The material was stored in plastic containers, transported to the laboratory, washed over 63 μm sieves and dried at room temperature [13]. The locations of individual sampling sites were precisely georeferenced via GPS and site-specific information is provided in Table 1 and Fig 1.

**Table 1:** Sample site information including *in situ* measurements of depth, pH, Total Dissolved Solids (TDS) and sea surface temperature recordings and sediment type (CS = Coarse Sand; FS = Fine sand; SM = Silty Mud; M = Mud).

STATION	LATITUDE	LONGITUDE	DEPTH (m)	pH	TDS (ppm [mg/l]	Temp (^O^C)	Sediment Type
1	6°23'59.73"	3°23'58.79"	20	6.9	8.4	29.8	CS
2	6°26'13.37"	3°23'58.59"	15	6.8	7.67	29.2	FS
3	6°27'32.27"	3°22'31.60"	20	6.5	7.83	29.1	FS
4	6°29'14.99"	3°24'12.62"	10	6.4	8.06	29.0	SM
5	6°31'3.77"	3°24'36.41"	5	6.9	1.31	29.2	M
6	6°32'43.78"	3°24'54.41"	7.5	6.6	1.22	29.2	M
7	6°34'32.38"	3°26'2.30"	3	6.6	0.96	27.5	M
8	6°35'24.84"	3°28'27.54"	4	6.5	0.31	27.3	FS
9	6°33'3.53"	3°27'44.75"	6	6.5	0.99	26.2	FS
10	6°30'56.07"	3°27'3.26"	7	6.6	1.01	27.8	M
11	6°28'27.27"	3°26'19.18"	7	6.4	0.98	27.7	M
14	6°28'6.16"	3°28'9.68"	12	6.5	1.76	27.7	M
15	6°27'42.21"	3°30'6.88"	12	5.8	1.68	26.3	SM
16	6°31'39.16"	3°33'8.84"	5	6.8	7.53	25.9	SM
17	6°29'44.65"	3°33'3.96"	4	6.7	8.21	25.1	SM
18	6°28'27.02"	3°33'14.44"	3	6.8	7.72	24.5	SM
19	6°30'40.31"	3°34'35.70"	4	6.7	8.72	24.9	SM
20	6°32'43.70"	3°34'34.85"	3	6.8	8.69	24.1	SM
21	6°32'5.01"	3°36'42.58"	3	6.6	7.65	24.2	SM
22	6°33'0.55"	3°38'25.59"	3	6.8	7.40	25.1	M
23	6°32'19.31"	3°41'49.52"	4	6.6	7.40	24.3	M
24	6°35'46.04"	3°41'44.34"	2	6.5	7.35	24.3	M
25	6°36'28.48"	3°45'37.70"	3	6.8	7.62	24.5	M
26	6°36'21.51"	3°49'37.21"	5	6.7	7.81	24.2	M

Foraminifera were then picked but not every sample yielded the standard amount of 300 specimens, as the abundance of benthic foraminifera varied from sample to sample. Benthic foraminifera were then identified to species level and individual taxa were documented by the Scanning Electron Microscopy (SEM), identified and assembled into a catalogue of taxa. For our analysis, living foraminifera were grouped with dead tests because our aim was to provide a general environmental and lagoon-wide data set useful in paleoecology. Our samples are thus time-averaged, and as such provide an effective means to compare changes recorded in the fossil record [14].

**Fig 2:**
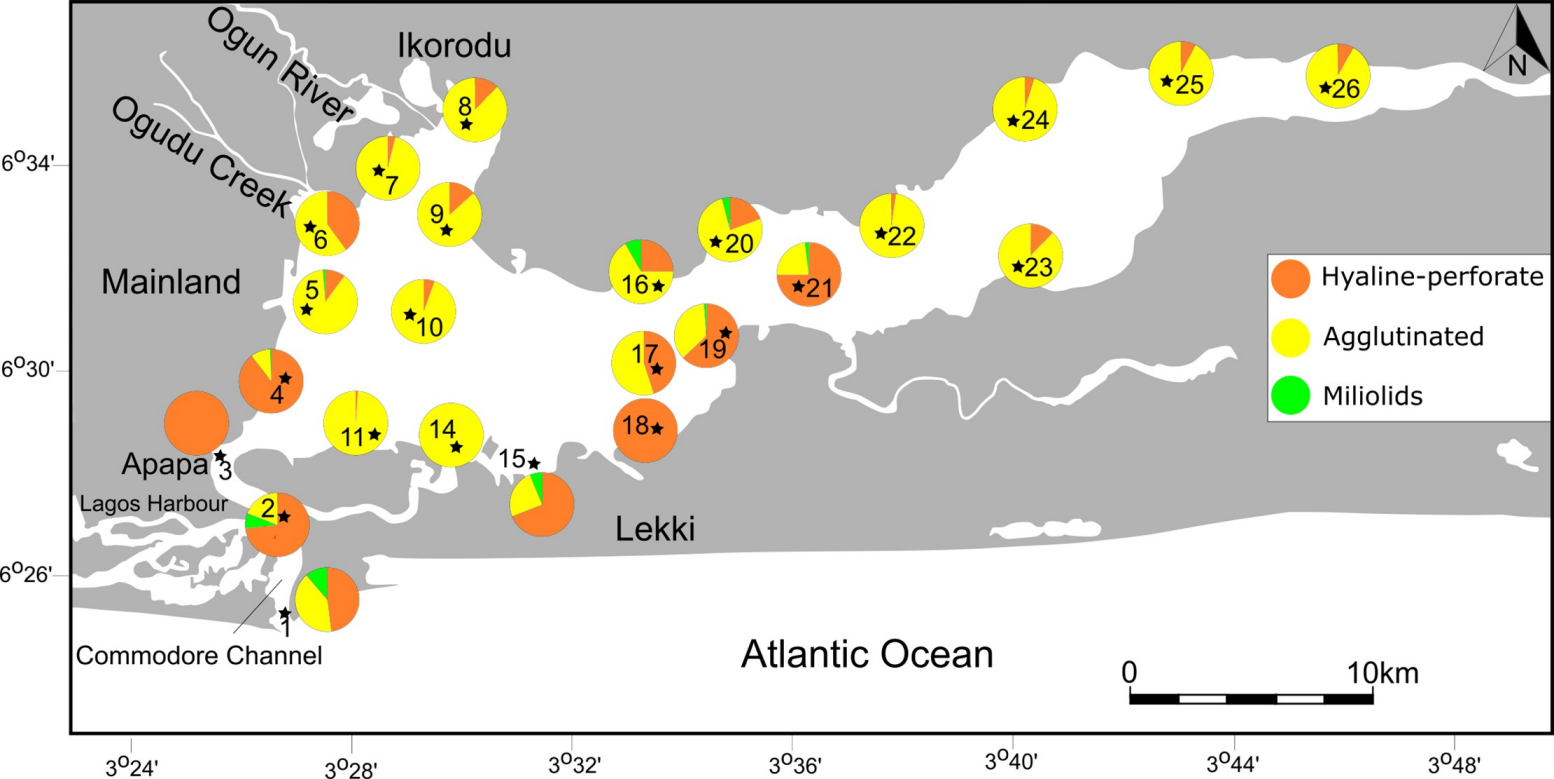
Percent abundances of agglutinated, hyaline-perforate and miliolid foraminifera across the Lagos Lagoon. Circle diagrams represent 100 percent of the total assemblage at the individual sites (for details see Table 2).

Individual species were counted and percent abundances were calculated for each taxon and for wall structural types (Table 2, Fig 2). Heat maps, showing the abundance of taxa, were then generated for selected species to document and analyze their distribution. The composition and structure of benthic foraminiferal assemblages were then analyzed from individual sites and further examined for diversity indices namely Fisher α, Shannon H (log base 10), Dominance D and total species richness.

**Table 2:** Quantitative faunal analyses of foraminiferal assemblages from the Lagos Lagoon, Southwest Nigeria, Gulf of Guinea.

Sample Stations	Taxa_S	Individuals	Dominance_D	Shannon_H	Fisher_α	% Hyaline	% Agglutinated	FN g^-1^
ST1	11	27	0.2016	1.937	6.92	48.1	40.7	7.7
ST2	23	68	0.1025	2.67	12.23	73.5	7.4	13.3
ST3	3	3	0.3333	1.099	0	100	0	0.6
ST4	8	189	0.5629	0.9499	1.694	89.4	10.1	61
ST5	12	237	0.4461	1.234	2.668	10.1	88.6	184.1
ST6	3	15	0.4489	0.9276	1.128	40	60	19.5
ST7	7	263	0.7499	0.6026	1.321	3.8	96.2	821.9
ST8	6	50	0.2928	1.485	1.78	12	88	33.4
ST9	7	68	0.6181	0.8894	1.957	13.2	86.8	34
ST10	7	258	0.5348	0.9502	1.327	5.4	94.6	345.8
ST11	6	302	0.6124	0.7636	1.061	1	99	523.4
ST14	2	2	0.5	0.6931	0	0	100	3.4
ST15	21	65	0.1569	2.352	10.76	69.2	24.6	16.7
ST16	23	213	0.3244	1.838	6.548	24.9	67	51.9
ST17	2	20	0.505	0.6881	0.5532	45	55	5.7
ST18	2	68	0.8153	0.3315	0.3864	100	0	44
ST19	8	84	0.3611	1.4	2.174	63.1	35.7	23.5
ST20	10	129	0.4766	1.232	2.531	19.4	76.7	43.9
ST21	13	196	0.4092	1.289	3.13	75	23	74.1
ST22	8	328	0.767	0.5517	1.48	2.1	97.9	1,012.3
ST23	9	335	0.5471	1.044	1.702	12.2	87.8	327.5
ST24	6	349	0.5596	0.9157	1.029	4.6	95.4	502.2
ST25	7	262	0.5438	0.9732	1.322	7.3	92.7	333.3
ST26	6	341	0.5776	0.8993	1.034	7.9	92.1	703.1

Numerical data include diversity and dominance indices of individual samples, percent abundances of hyaline and agglutinated foraminifera specimens, and foraminiferal number (FN) per gram sediment.

To determine the structure in our foraminiferal data set, statistical analyses (Cluster, Principal Component [PCA] and Detrended Correspondence Analysis [DCA]) were carried out using the PAST3 software [15]. These techniques group samples with similar faunal assemblages and reveal a typology of environmental signatures embedded in foraminiferal assemblages. PCA and DCA are helpful in a multivariate analysis to structure and visualize larger data sets by reducing a large number of variables to a few linear combinations (principal components). In addition, the foraminiferal number (FN) per gram of treated sediment sample was counted for all the sites [16–18].

A Hanna HI 9813-6N multi-parameter sensor probe-device was used to record the physico-chemical and environmental data including depth, temperature, pH and total dissolved solids (TDS = combined content of all inorganic and organic substances). Environmental data recordings were conducted at the water surface and are provided in Table 1). For species identifications we have applied the concepts of the nearest complete faunal studies from Mikhalevich [19, 20], Debenay and Basov [21], Debenay and Redois [22], Langer et al. [13, 23] and Fajemila and Langer [24, 25], Thissen and Langer [26], Langer et al. [27], Hayward et al. in press [28].

## Results

### Physico-chemical measurements

Surface water temperature measurements revealed a range between 29.8 and 24.1°C across the lagoon. Temperatures were found to be generally high (>29°C) around the entrance channel and along the southwestern shore (ST1-6). Towards the northwestern sector, near the Ogun River, and in the central parts of the lagoon, temperatures drop to ~26°C and become successively lower towards the easternmost sector (>24°C).

Ph values were found to be largely homogenous across the lagoon and range between 5.8 and 6.9 (see also [29]). The lowest values were recorded in the northwestern sector near the Ogun River mouth (<6.7). Highest values were recorded in the entrance channel and around Lagos harbor (>6.8, ST1, ST2), where marine waters enter the lagoon system. Medium and high values were recorded at a few selected sites in the central part of the lagoon (Table 2).

Total dissolved solids were found to range between 0.31 to 8.72 mg/l. Highest values (>7.35) were recorded around the entrance channel and along the southwestern shore (ST1-4) and in the murky waters of the eastern sector (ST16-ST26). Medium values were recorded in the western lagoon, and lowest values were found in the northwestern area (ST7-9) near the mouth of the Ogun River.

The physicochemical measurements, however, only provide a snapshot of environmental conditions at the time of collection and do not account for seasonal variations related to the wet and dry season and runoff from the Ogun River. As such they are of limited value for the analysis of total assemblages.

### Composition of foraminiferal assemblages

A total of 3872 benthic foraminifera specimens were picked and identified to species level whenever possible. This resulted in the identification of 42 species belonging to 25 genera. The foraminiferal assemblage comprises 10 porcelaneous, 22 hyaline perforate and 10 agglutinated taxa. Agglutinated foraminifera are the dominant group with an abundance of 77.9%, while the hyaline-perforate species make up 21.1% of the total assemblages. The remaining 1% belongs to the porcelaneous taxa (Table 2).

Agglutinated foraminifera dominate the foraminiferal assemblages over large parts of Lagos Lagoon (Fig 2, Table 2) and constitute ~90% of the total assemblage at many sites in front of the Ogun River (ST5, ST7, ST8, ST9, ST10, ST11), make up more than 67% along the northern shore (ST16, ST20), and comprise mostly more than 90% of the fauna in the easternmost sector of the lagoon (ST22-ST26).

Foraminifera with a hyaline perforate test are dominant around Lagos Harbor and the Commodore Channel (ST1- ST4), where the lagoon empties its waters into the Atlantic, and marine waters mix with brackish lagoonal waters. High abundances of perforate foraminifera where also found along the lagoonal shore off Lekki.

Miliolid foraminifera are generally rare within the entire lagoon and are mostly represented by species of the genus *Quinqueloculina*. The abundance of porcelaneous species corresponds to only 1% of the total population of benthic foraminifera of the Lagos Lagoon. Highest occurrences of miliolids were recorded near the entrance channel (<19%), where open ocean and brackish waters mix and salinity values are commonly higher than in the lagoon. Miliolids are absent in the easternmost parts of the lagoon and near the mouth of the Ogun River, where low salinity and freshwater conditions are predominant for most of the year. The distribution of miliolids, hyaline-perforate, and agglutinated foraminifera neither covaries with pH nor with lagoon surface water temperature recordings (see Table 1).

**Fig 3:**
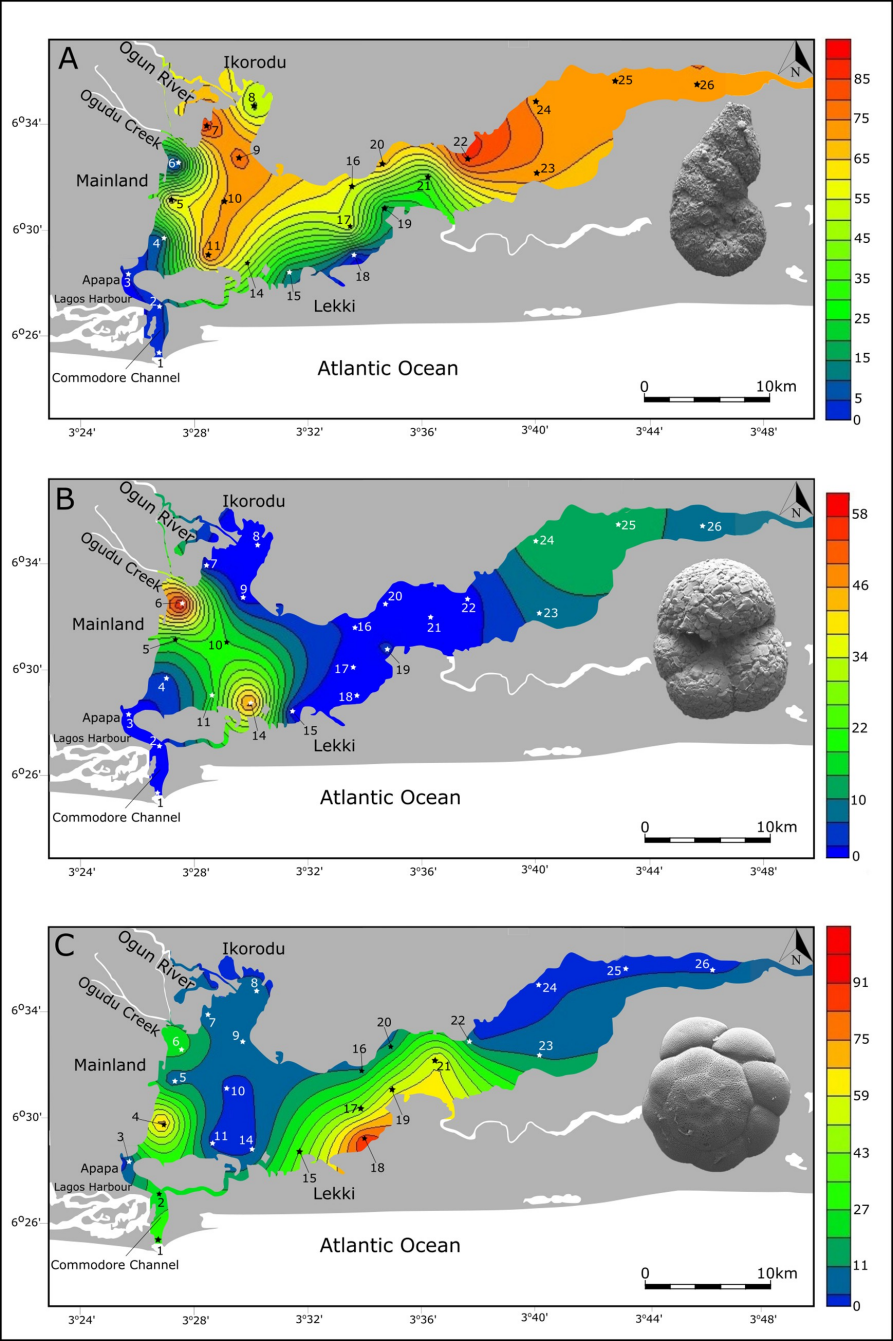
Heat maps showing color-coded percent abundances with interpolated distribution contours for A.) *Ammotium salsum*, B.) *Trochammina* sp. 1. and C.) *Ammonia aoteana*, within the Lagos Lagoon, Nigeria.

Agglutinated species of the genus *Ammotium* dominate the assemblages within the lagoon at many sample sites (Table 2 and Fig 3A) and account for about 62.8% of the total population of benthic foraminifera. *Ammotium salsum* alone frequently reaches abundance levels of more than 50% in some samples (ST5, ST7, ST10, ST11, ST16, ST20, ST22, ST23, ST24, ST25, ST26). It is particularly abundant in shallow waters (< 7m) in the northwestern part of the lagoon near the mouth of the Ogun River (> 65%) and in the eastern areas of Lagos lagoon. Both areas are characterized by low salinity but salinity varies with the season (0–10‰). Along the densely populated western shore, around the harbor and other industrial structures, and in southern shallow waters off Lekki, abundance values drop substantially and commonly range below 25%. Within the deeper and highly saline Commodore entrance channel, percent abundance values of *Ammotium salsum* display lowest values and range below 5%.

The agglutinated taxon *Trochammina* sp. 1 is also fairly abundant (Fig 3B) and frequently constitutes more than 10% to the population at many sample sites (ST5, ST6, ST10, ST11, ST24, ST25). The species occurs throughout the Lagos Lagoon, its abundance maxima, however, are antagonistic to those of most other agglutinated taxa and differ significantly from those of *Ammotium salsum*. The species reaches its highest total abundance values at site ST6 and ST14 (60% and 50%). Site ST6 is located south of dense mangrove forests at the mouth of Ogudu Creek, where sewage, industrial wastewater and heavy metal concentrations accumulate. *Trochammina* sp. 1 is abundant where most other agglutinated taxa (*Ammotium salsum*, *Miliammina fusca*, *Ammobaculites exiguus*, and *Textularia* sp.) have minimal or rare occurrences.

*Ammonia aoteana* dominates the group of hyaline-perforate foraminifera in the Lagos Lagoon sample material. It constitutes 14.1% of the total population of benthic foraminifera recovered and percent abundances at individual sites range between 0 and 90%. It is prominent at some sites along the densely populated western part of the lagoon (<20%, ST4, ST6), in the entrance of the Commodore Channel (ST1 and ST2), and strikingly abundant in the shallow central parts SE of Lekki (ST15, ST17, ST18, ST19, ST21; Fig 3C). Abundance peaks of *Ammonia aoteana* were mostly recorded in shallow waters (3-7m) with highest value of 90% at sample station ST18 (3m). The distribution of the second species of *Ammonia*, *Ammonia convexa*, matches the distribution of *Ammonia aoteana*, but percent abundance values of *A*. *convexa* are generally low and rarely exceed 10%. *Ammonia convexa* was absent at site ST3, ST7, ST11, ST14 and ST17. In total, the species accounted for about 4% of the population of all benthic foraminifera recovered.

*Miliammina fusca*, a miliolid foraminifer with an agglutinated test, was recorded in low numbers at a few sites within the lagoon. Its presence, however, is to be restricted to the northwesternmost area near the Ogun River mouth (ST7-ST9) and to two sites in the low salinity areas in the eastern sector of Lagos Lagoon (ST22-ST23).

Miliolid foraminifera are generally rare in the lagoon and contribute ~1% to the total assemblage. Highest abundances (~19%) were recorded in close proximity to the Atlantic entrance (ST1, ST2) and moderate values were recorded at site ST15, ST16 and ST19 (<8%). At all other sites, miliolids are extremely rare and occur only sporadically (Fig 2 and Table 2 and S1 Appendix).

Only 4 individuals of larger symbiont-bearing foraminifera were recovered from the entire lagoon material. They belong to *Pararotalia* and *Amphistegina*, and constitute only ~ 0.1% of the total assemblage. The few individuals recovered were recorded at sample stations ST10, ST15, ST16, three sites that receive marine water via the main entrance channel and the Five Cowries Creek.

### Foraminiferal numbers

**Fig 4:**
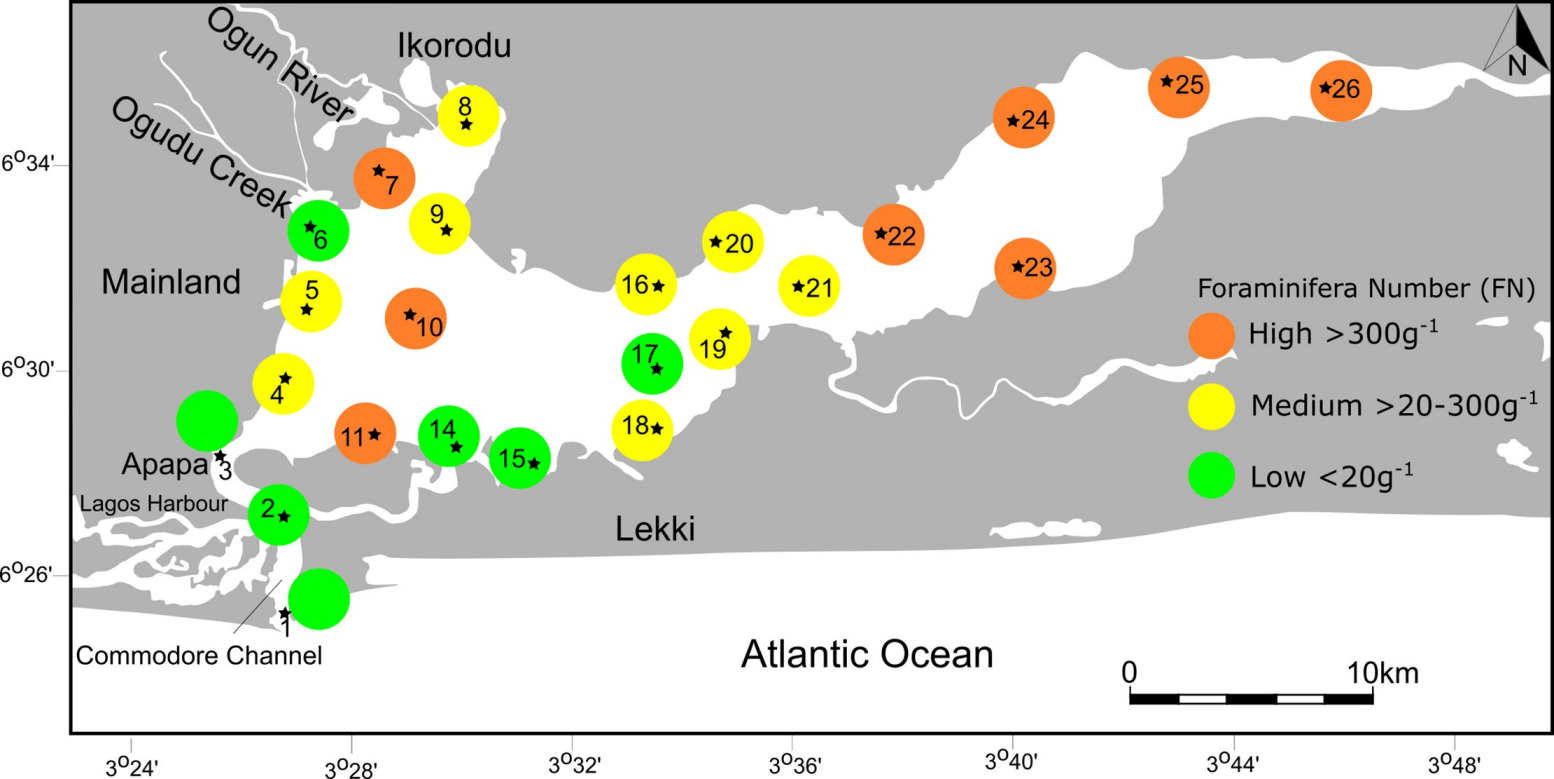
Number of foraminifera per unit gram of sediment (FN) in the Lagos Lagoon. Note consistently high values in the eastern sector medium to high values along the continuation of the Ogun River outflow.

The number of foraminifera per gram sediment (FN) varies substantially among individual samples and ranges from 0.6 to more 1000 g^-1^ (Table 2). Highest values (>300) were recorded in the eastern sector (ST22-ST26), at the northwesternmost end near the mouth of the Ogun River (ST7), and in the central and southern part of the lagoon (ST10, ST11). Low and medium values (>20–200 g^-1^) were found at two sites on the western lagoon shores (ST4, ST5), in the northwestern area (ST8, ST9), and at the transition from the central lagoon to the eastern sector (ST 16, ST 18-ST21). Lowest values (<20 g^-1^) were found at the mouth and in the entrance channel of the lagoon (ST1-ST3), at the mouth of the Ogudu Creek (ST6), and at two sites along the southern shore of Lagos Lagoon (ST14, ST15). The FN covaries neither with pH nor with surface water temperature recordings (see Table 2, Fig 4).

### Diversity

**Fig 5:**
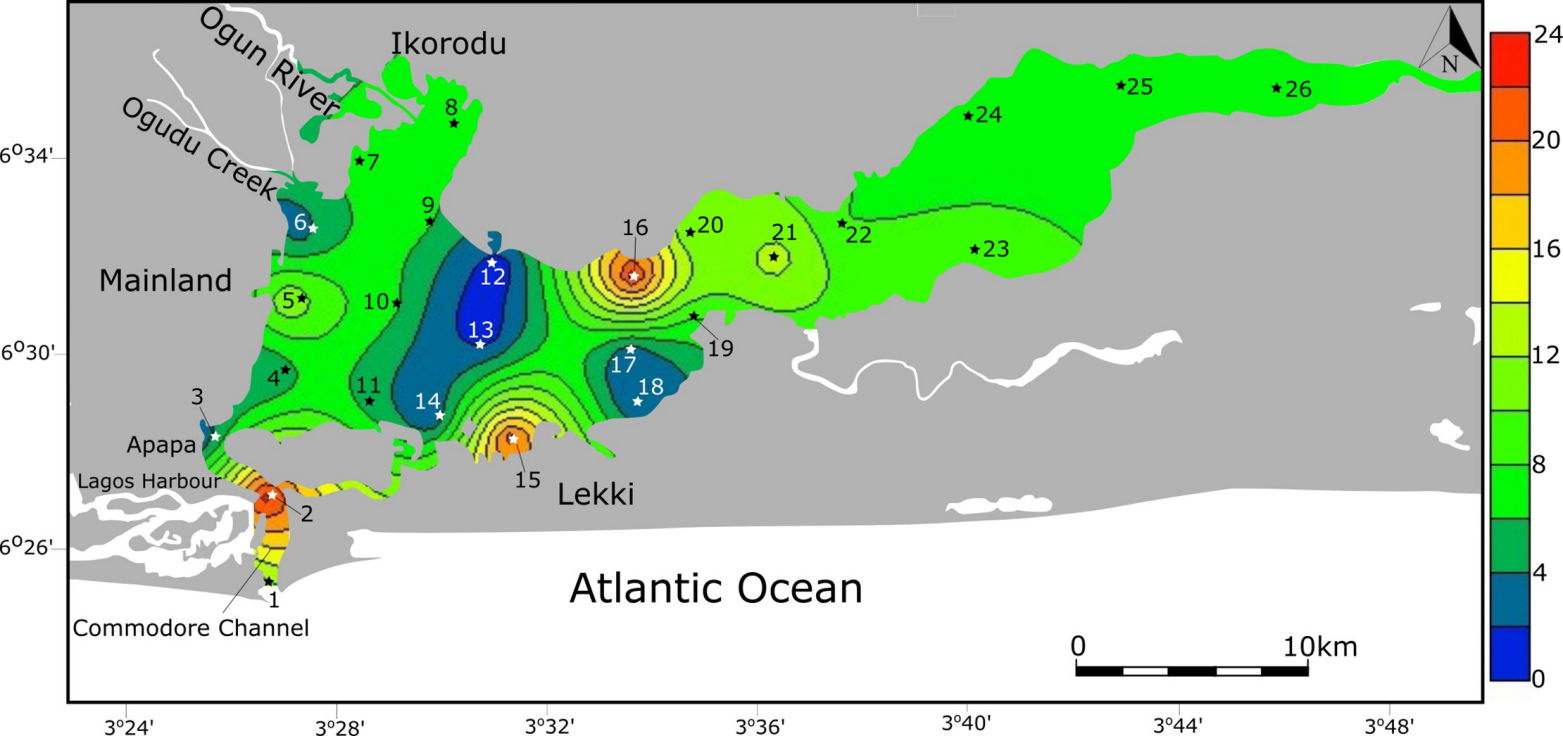
Map summarizing color-coded species richness values at each sampling location.

The number of benthic species of foraminifera present in Lagos Lagoon was counted at all sites and the results are illustrated in Fig 5. Species richness ranges between 2 and 23 across Lagos Lagoon. Highest values were recorded near Lagos Harbor (ST2, 23 species), were lagoonal waters mix with the adjacent Atlantic Ocean, on the southern shore near Lekki (ST15, 21 species), and at site ST16 (23 species) near Baiyeku. Species richness in the eastern sector of the lagoon (ST22-ST26) was considerably lower and ranges from 6–9. In the northwestern sector, near the mouth of the Ogun River (ST7-ST10), the number of taxa was also recorded to be low and ranges from 6–7. Particularly low species richness values were noted along the western shore near the outlet of Ogudu Creek and in Lagos Harbor (ST6, ST3; 3 species each), and at sites ST14, ST17 and ST18 (2 species), which possibly result from either intense pollution or dredging activities taking place in these localities.

Fisher α diversity values range between 0 and 12.23 in the Lagos samples. The highest Fisher α values were recorded ST1, ST2, ST15, and ST16. The lowest Fisher α index values were noted at ST3, ST14, ST17 and ST18. The high and low Fisher α recordings are congruent with maximum and minimum species richness values recorded at these sites. Shannon (H) values vary between 0.33 and 2.67; the highest values were observed at ST1 (1.937), ST2 (2.67), ST 15 (2.352), and ST 16 (1.838). The lowest Shannon (H) values were recorded at ST7, ST14, ST17, ST18, and ST22. Dominance values range between 0.1 and 0.81. Shannon (H) and Dominance index values are in general accordance with species richness values. Neither species richness nor Fisher α index values covary with pH or surface water temperature recordings (see Table 1).

### Cluster analysis

Cluster analysis (R- and Q- mode), analyzing the similarity, composition and abundance of foraminifera at individual sample stations were performed on the 13 most abundant benthic foraminifera recorded throughout the lagoon (Table 3). The 13 species selected represent 98.3% of the total population of all foraminifera counted. Samples ST3 and ST14 were excluded from the Q-mode cluster analysis, because they did contain sufficiently high numbers of specimens (<4).

**Table 3:** Thirteen (13) most frequent and abundant benthic foraminifera from the lagoon.

SST	A	B	C	D	E	F	G	H	I	J	K	L	M
**ST1**	0	1	9	1	0	1	0	0	1	1	0	7	0
**ST2**	0	6	16	1	0	6	6	0	2	8	2	3	0
**ST3**	0	0	0	0	0	1	0	0	0	1	0	0	0
**ST4**	0	24	139	12	0	1	4	0	0	0	0	0	7
**ST5**	14	4	13	151	2	0	4	0	2	0	1	0	43
**ST6**	0	2	4	0	0	0	0	0	0	0	0	0	9
**ST7**	14	1	9	227	6	0	0	5	0	0	0	0	1
**ST8**	6	4	2	24	8	0	0	6	0	0	0	0	0
**ST9**	3	3	5	53	2	0	0	1	1	0	0	0	0
**ST10**	13	8	5	182	2	0	0	0	0	0	0	0	47
**ST11**	19	1	2	231	3	0	0	0	0	0	0	0	46
**ST14**	0	0	0	1	0	0	0	0	0	0	0	0	1
**ST15**	0	9	21	9	0	1	4	0	1	0	1	5	0
**ST16**	17	7	21	117	3	6	7	0	3	3	3	10	4
**ST17**	0	0	9	11	0	0	0	0	0	0	0	0	0
**ST18**	0	7	61	0	0	0	0	0	0	0	0	0	0
**ST19**	8	4	47	15	5	2	0	0	0	0	0	0	2
**ST20**	9	3	15	87	0	4	0	0	2	1	4	3	0
**ST21**	2	21	117	39	2	1	0	0	0	7	1	2	0
**ST22**	26	4	3	286	3	0	0	3	0	0	0	0	1
**ST23**	15	5	29	244	6	0	7	2	0	0	0	0	26
**ST24**	28	8	8	255	3	0	0	0	0	0	0	0	47
**ST25**	19	14	5	189	2	0	0	1	0	0	0	0	32
**ST26**	31	20	7	255	1	0	0	0	0	0	0	0	27

They correspond to 98% of the entire population of the foraminifera counted: **A**- *Ammobaculites exiguus*; **B**- *Ammonia convexa*; **C**- *Ammonia aoteana*; **D**- *Ammotium salsum*; **E**- *Ammotium* sp.1; **F**- *Hanzawaia* cf. *H*. *nipponica*; **G**- *Cribroelphidium mirum*; **H**- *Miliammina fusca*; **I**- *Neoeponides* sp. 1; **J**- *Nonion fabum*; **K**- *Quinqueloculina seminulum*; **L**- *Textularia* sp. 1; **M**- *Trochammina* sp. 1; **ST**-Sample Station.

**Fig 6:**
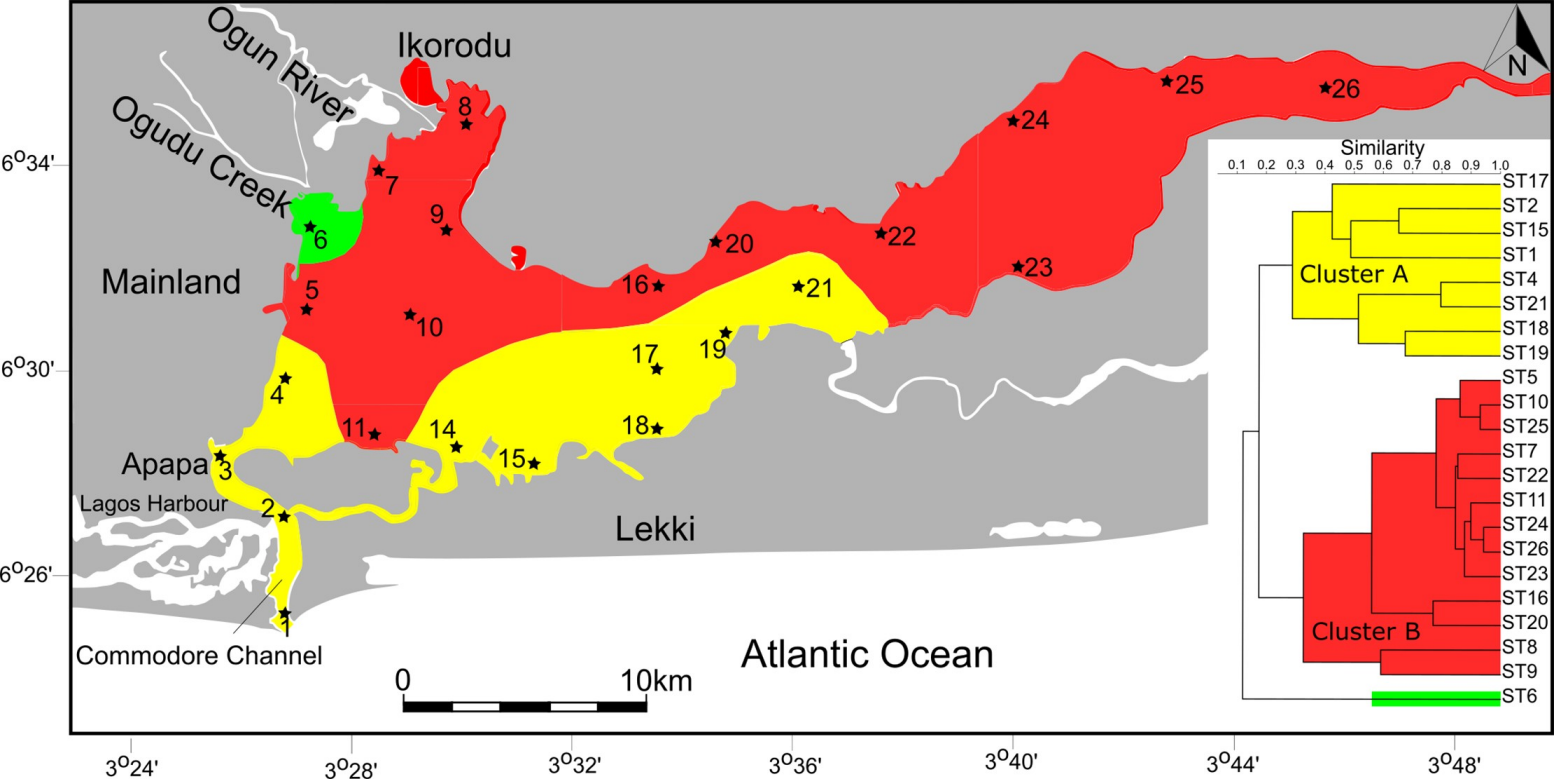
Q-mode cluster analysis and distribution of cluster groups across Lagos Lagoon.

Cluster analysis (Q-mode), comparing the composition and abundance of foraminifera assemblages from all sample sites, revealed the presence of two units (cluster A-B, Fig 6) and an outlier at ST6. The dendrogram shows that individual clusters occupy different sectors of Lagos Lagoon that are characterized by specific environmental conditions. Cluster A comprises the sample sites that are located near the entrance channel (ST1-ST4) and all sample stations that are located on the southern lagoon shores in the central part of the lagoon (ST15-ST21). Cluster B covers the northwesternmost sites where freshwater from the Ogun River drains fan-like into the lagoon (ST5, ST6-ST11), sites along the northern lagoon shores (ST16, ST20) and all sites located in the eastern lagoon sector (ST22-ST26). The outlier site ST6 is situated at the mouth of the Ogudu Creek, where saltgrass meadows and mangrove trees flourish along the lagoon coastline.

**Fig 7:**
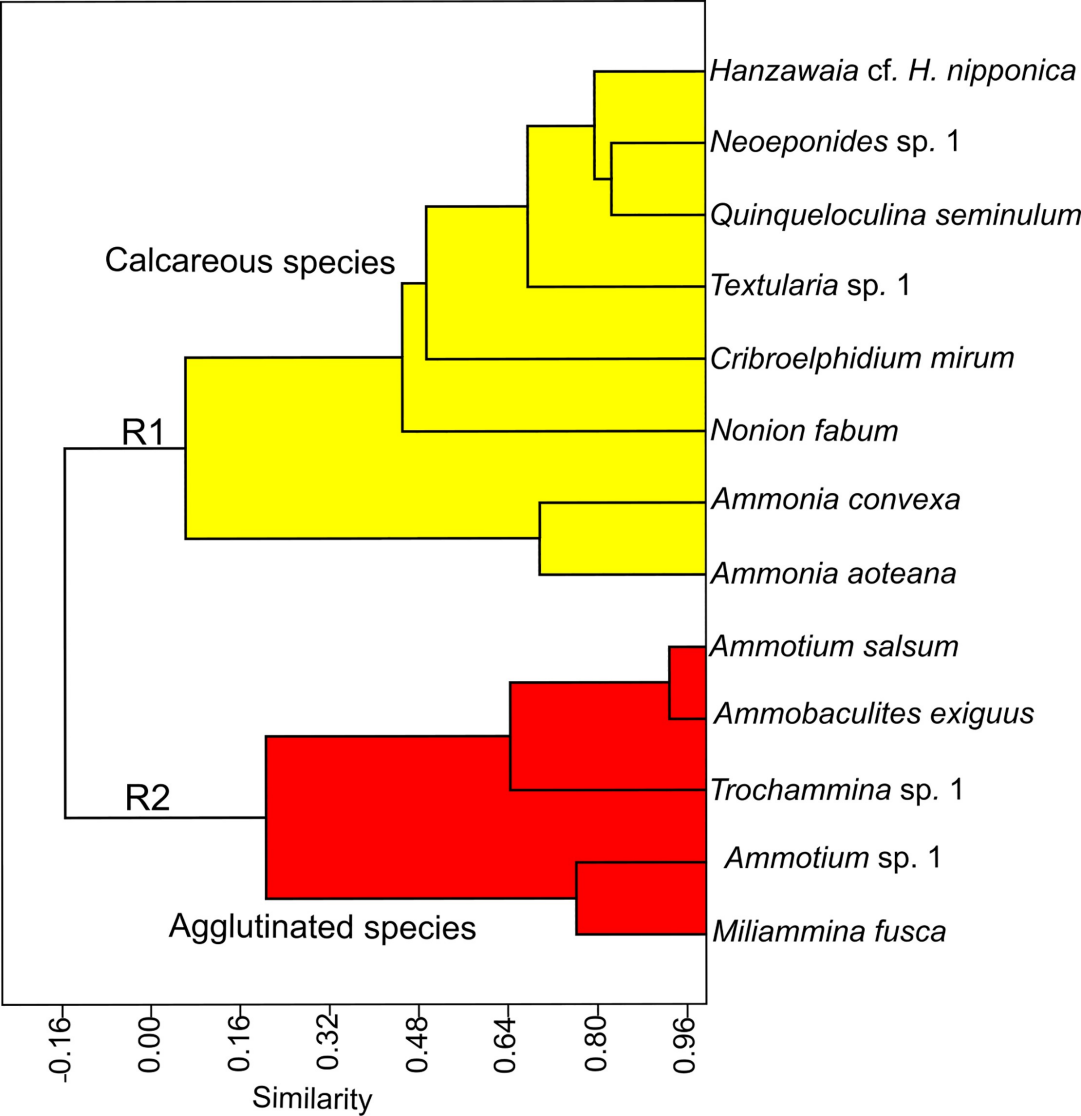
Species dendrogram produced by the R-mode cluster analysis using the correlation coefficient matrix. Note that R-mode clusters reflect test wall types (agglutinated versus hyaline-perforate/porcelaneous).

The R-mode analysis resulted in a dendrogram that also revealed two major clusters (Clusters R1 and R2, Fig 7). Cluster R1 comprises a total of 8 species and contains exclusively taxa with hyaline-perforate or porcelaneous wall structure types. Species characterizing cluster R1 have their highest abundances along the industrial areas in southwestern lagoon area, in the Commodore entrance channel and at sample stations that are located on the southern lagoon shores and in the central parts of the lagoon. The distribution recordings of species contained in cluster R1 show that these taxa favor marine and avoid low salinity, brackish or freshwater conditions.

Cluster R2 comprises a total of 5 species and is dominated by taxa with an agglutinated wall structure (*Ammotium*, *Ammobaculites*, *Trochammina)*. Cluster R2 is associated with low-salinity, brackish water and freshwater conditions. Among the species of cluster R2, we also find *Miliammina fusca*, *a* species that is often the last identifiable species to survive under marginal marine and low salinity conditions.

### Principal component analysis

**Fig 8:**
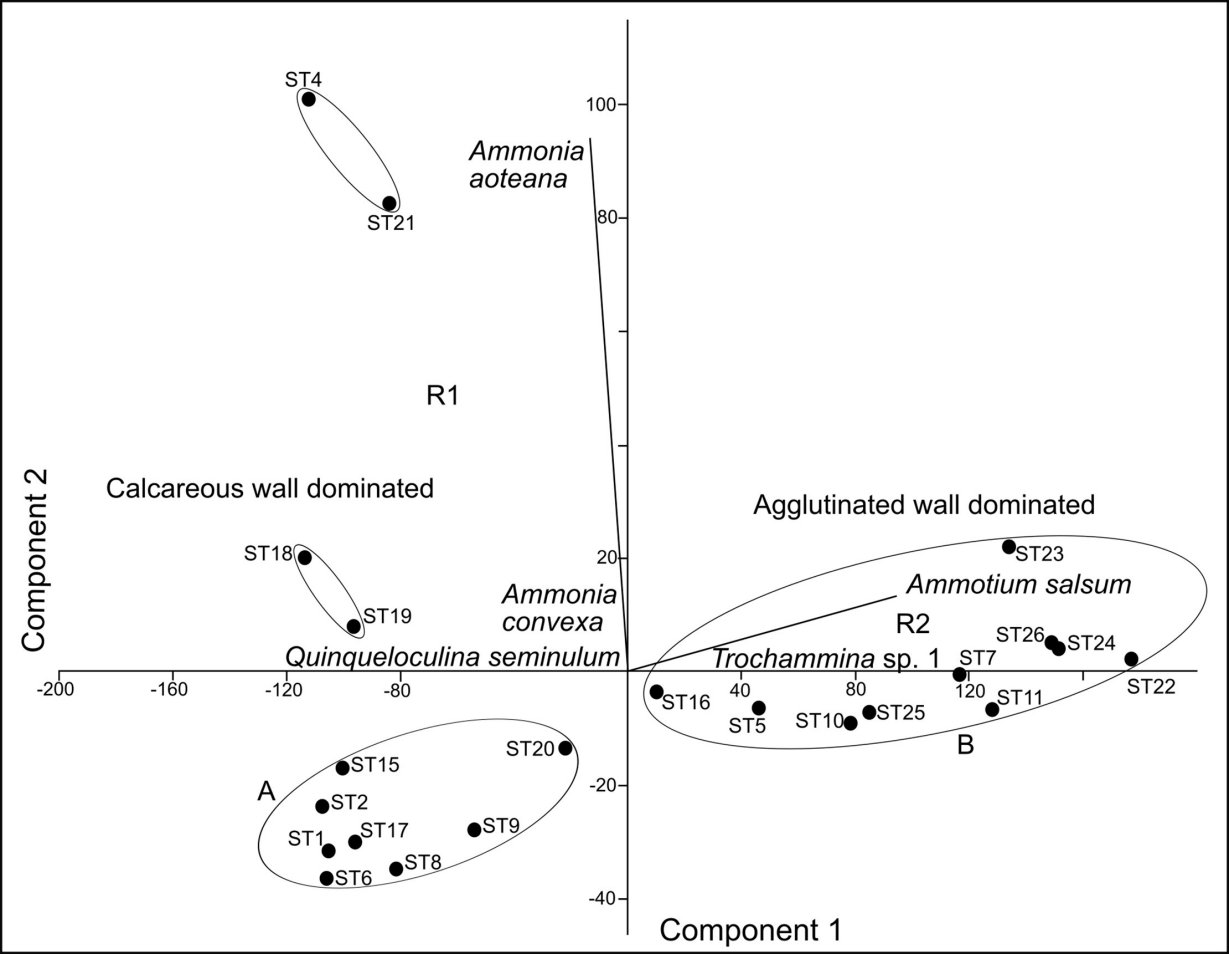
PCA of the foraminiferal fauna of the Lagos Lagoon showing principal components 1 and 2 (A, B and R1, R2 refer to the assemblages defined in Figs 6 and 7).

Principal Component Analysis (PCA) is a dimension-reduction tool that can be used to minimize a large set of variables, which helps to describe and classify our extensive foraminiferal occurrence dataset. Like the cluster analysis, the principal component analysis was conducted with the 13 most abundant benthic taxa. The species are shown as vectors and their lengths represent the importance of individual species as calculated by their eigenvalues. It revealed two major groups, that are largely separated by structure (Fig 8). The agglutinated group, which is prevalent in the eastern sector of the lagoon, occupies the B ellipsoid. The *Ammotium salsum* vector is strongly related to those sample stations and includes all sample sites from ST22 to ST26. The second vector includes the sites that are dominated by specimens of the hyaline-perforate genus *Ammonia* (ST4, ST21, ST18, ST19, ST15, and ST2). The sites occupy the densely populated western part and the central southern shores of the lagoon.

### Detrended correspondence analysis (DCA)

**Fig 9:**
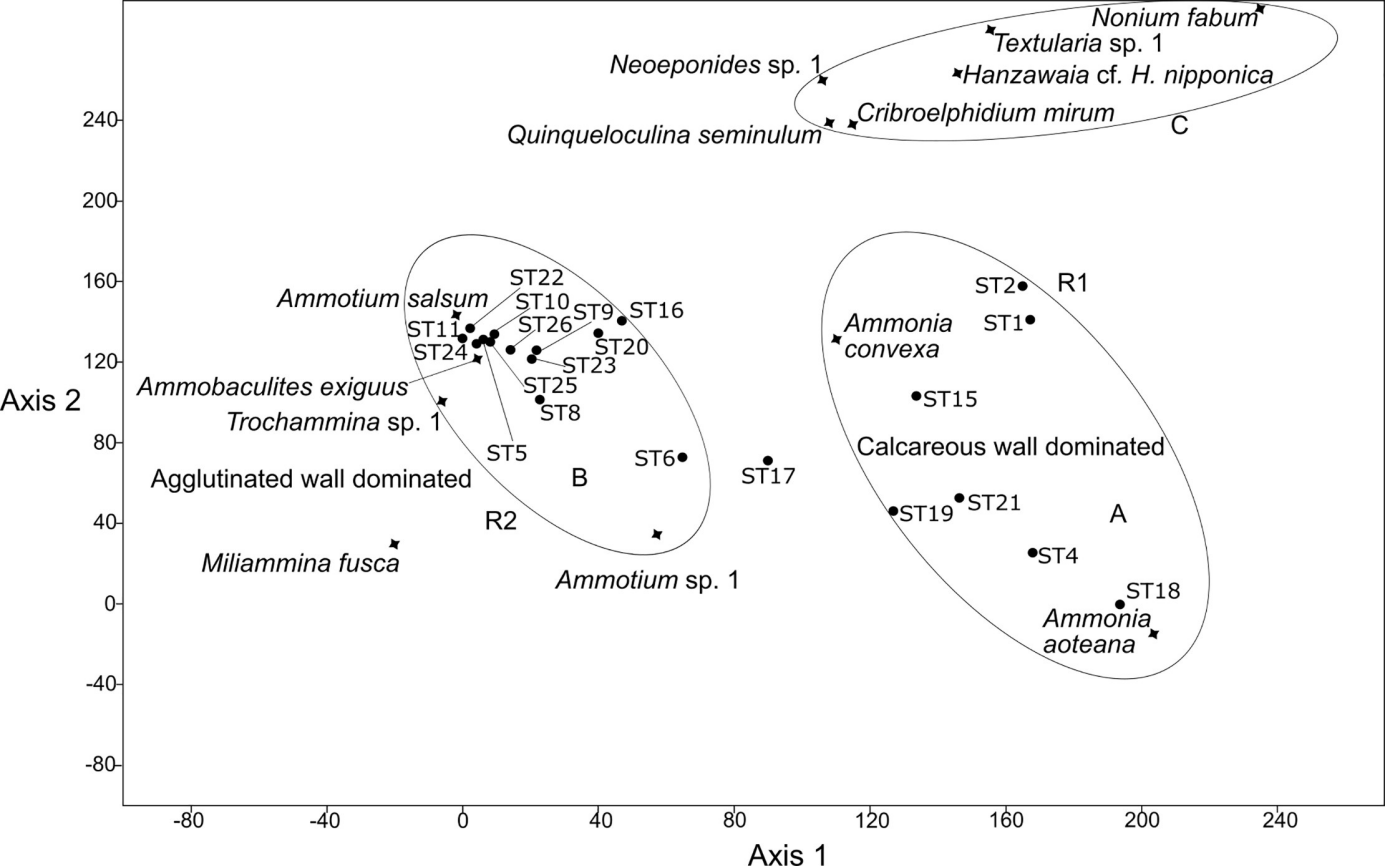
Detrended correspondence analysis of the benthic foraminiferal species recovered from the Lagos Lagoon (A, B and R1, R2 refer to the assemblages defined in Figs 6 and 7).

The detrended correspondence analysis revealed three groups (A-C, Fig 9) that are mainly separated by the wall structural types and sample sites. Group A is dominated by *Ammonia aoteana* and *Ammonia convexa*, two species that have abundance maxima in the southwestern areas of Lagos Lagoon (see also Fig 3C). Group B is dominated by *Ammotium salsum* and other species with agglutinated tests (*Trochammina* sp. 1, *Ammotium* sp. 1, *Ammobaculites exguus*). *Miliaminna fusca* is also associated to group B. Group C contains a mixture of heterogenous and mostly rare species with different types of wall structures from different genera (e.g., *Hanzawaia*, *Textularia*, *Nonion*, *Elphidium*, *Quinqueloculina*, Fig 9). Members of group C are typical marine taxa, and their distribution in the lagoon is mainly restricted to the area that connects the Atlantic Ocean with Lagos Lagoon (ST1-ST3) and the southwestern and central area, that receives marine waters via the Five Cowrie Creek (ST15, ST16, ST19-ST21).

## Discussion

**Fig 10:**
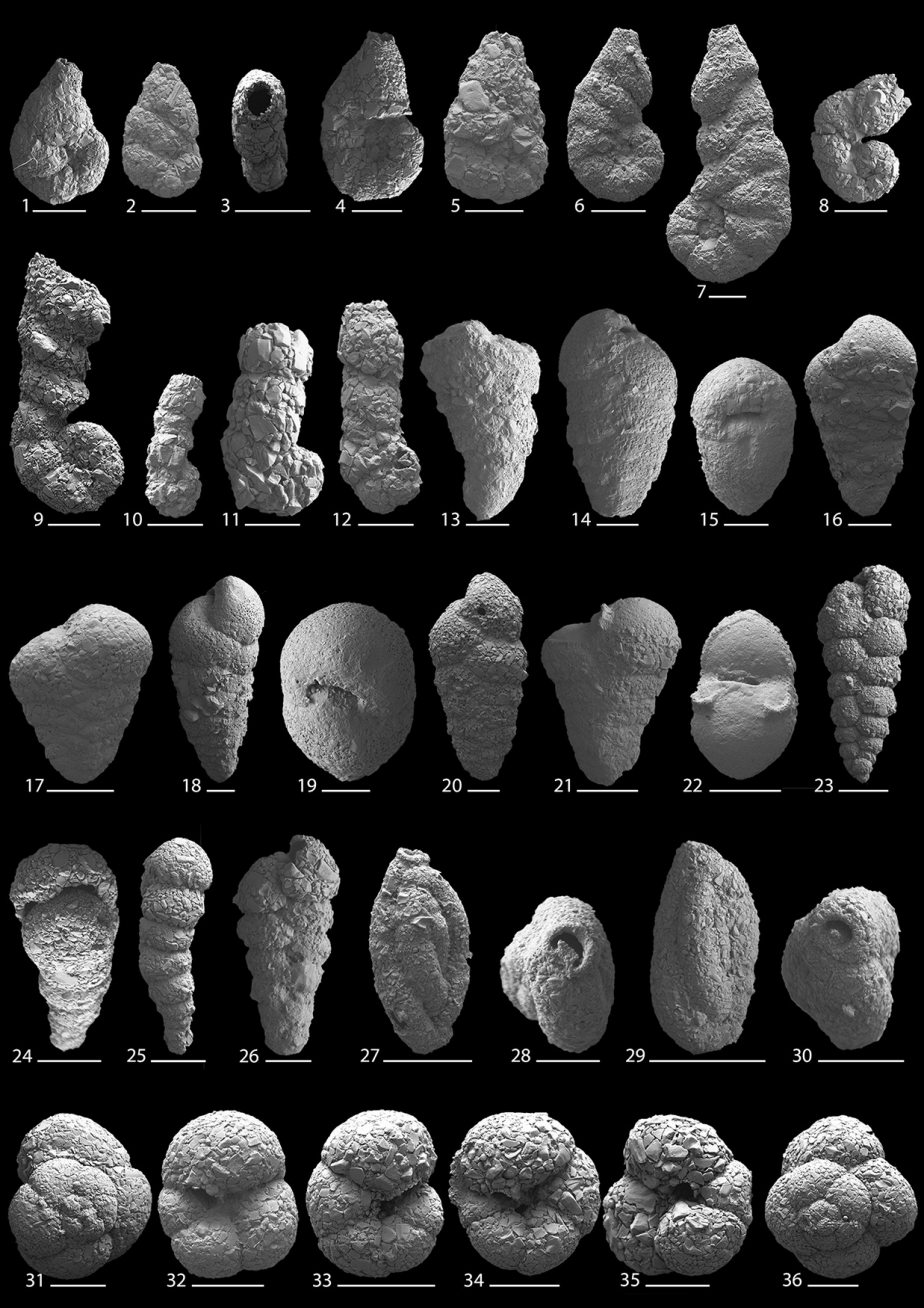
Scanning electron micrographs of benthic foraminifera from the Lagos Lagoon: 1–7. *Ammotium salsum* (Cushman and Brönnimann, 1948); scale bar for Fig 10.2 is 200 μm; **8, 9**. *Ammotium* sp. 1; **10–12.** *Ammobaculites exiguus* Cushman and Brönnimann, 1948; **13**. *Textularia candeiana* d’Orbigny, 1839; **14–17**. *Textularia* sp. 1; **18–20**. *Textularia* sp. 2; **21, 22**. *Siphotextularia* sp. 1; **23–26**. *Caronia exilis* (Cushman and Brönnimann, 1948); scale bar for Fig 10.26 is 50 μm; **27–30**. *Milliamina fusca* (Brady, 1870); **30–36**. *Trochammina* sp. 1. Scale bar is 100 μm for all magnifications.

**Fig 11:**
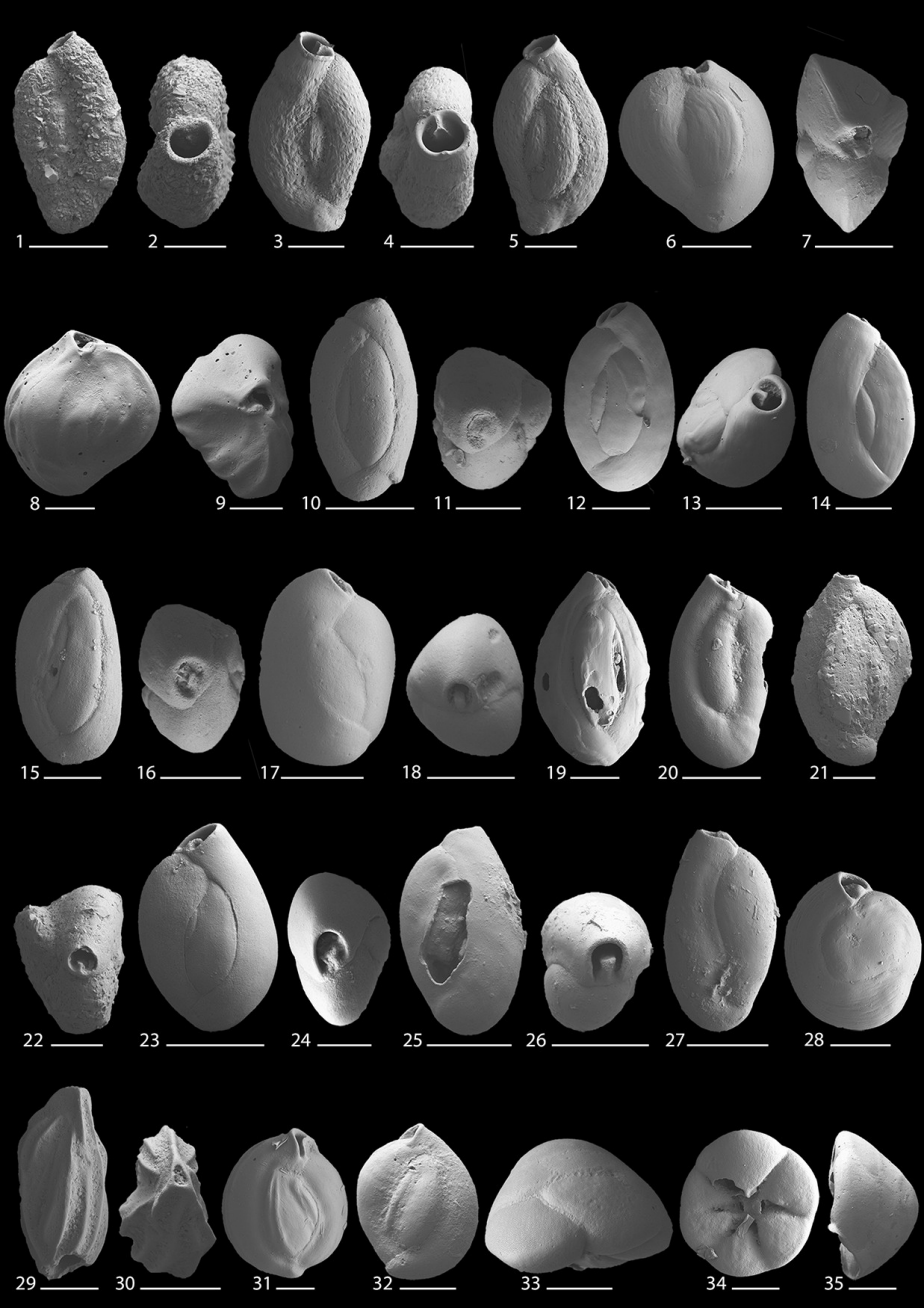
Scanning electron micrographs of benthic foraminifera from the Lagos Lagoon: 1, 2. *Trilocularena patensis* Closs, 1963; **3–5**. *Quinqueloculina debena*yi Langer, 1992; **6–9**. *Quinqueloculina* cf. *Q*. *cuvieriana* d’Orbigny, 1839; **10, 11**. *Quinqueloculina seminulum* Linné, 1758; **12–14**. *Quinqueloculina* cf. *Q*. *seminulum* Linné, 1758; **15, 16**. *Quinqueloculina* cf. *Q*. *vandiemeniensis* Loeblich and Tappan, 1994; **17, 18**. *Quinqueloculina* sp. 1; **19**. *Quinqueloculina* sp. 2; **20**. *Quinqueloculina* sp. 3; **21, 22**. *Quinqueloculina* sp. 4; **23–27**. *Triloculina* cf. *T*. *verspertilo* Zheng, 1988; **28**. *Pseudotriloculina* sp. 1; **29, 30**. *Edentostomina* sp. 1; **31, 32**. *Miliolinella* sp. 1; **33–35**. *Neoeponides* sp. 1. Scale bar is 100 μm for all magnifications, and 50 μm for Figs 11.2, 11.11, 11.15, 11.16, 11.24, 11.31, and 11.32.

**Fig 12:**
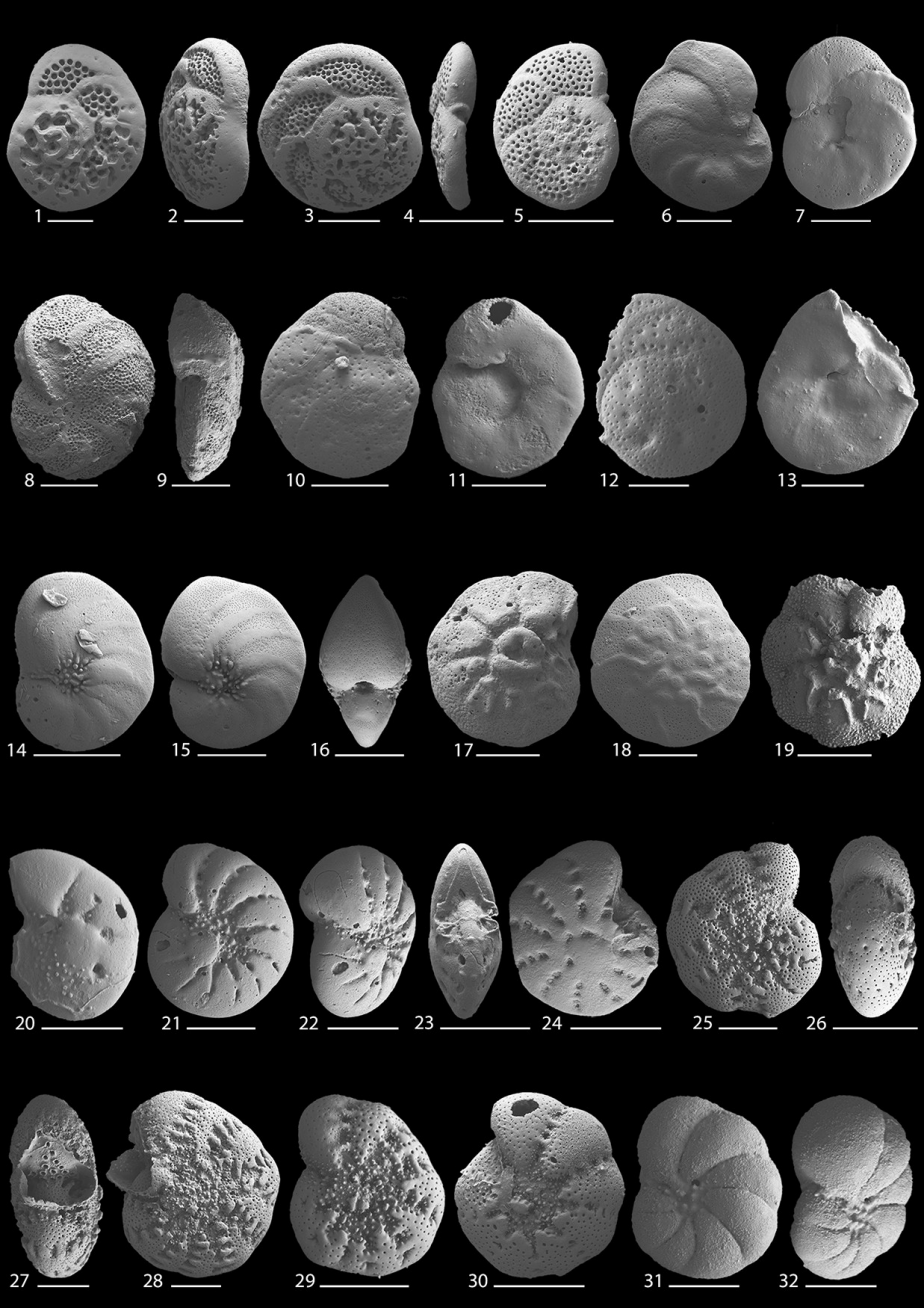
Scanning electron micrographs of benthic foraminifera from the Lagos Lagoon: 1–5. *Rosalina* cf. *R*. *orientalis* (Cushman, 1925); **6–9**. *Hanzawaia* cf. *H*. *nipponica* Asano, 1944; **10–13**. *Planulina* sp. 1; **14–16**. *Nonion fabum* (Fichtel and Moll, 1798); **17–19**. *Pararotalia sarmientoi* (Redmond, 1953); **20–24**. *Elphidium* sp. 1; **25–30**. *Cribroelphidium mirum* Langer and Schmidt-Sinns, 2006; **31, 32**. *Porosononion* sp. 1. Scale bar is 100 μm for all magnifications and 50 μm for Figs 12.10–12.13, 12.31 and 12.32.

**Fig 13:**
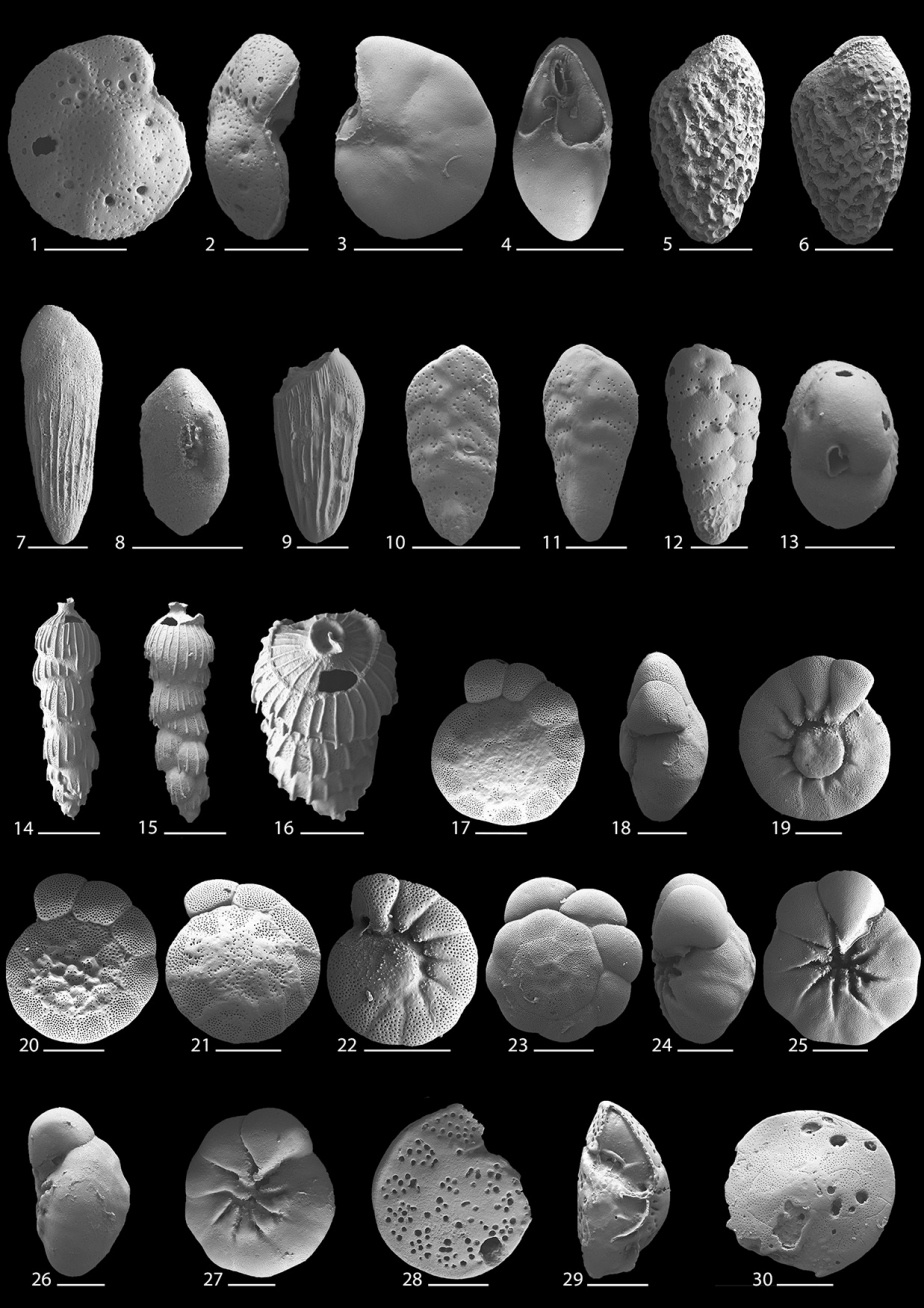
Scanning electron micrographs of benthic foraminifera from the Lagos Lagoon: 1, 2. *Rotorbis*? sp. 1; **3, 4**. *Globocassidulina*? sp. 1; **5, 6**. *Bolivina* cf. *B*. *persiensis* Lutze, 1974; **7–9**. *Bolivina striatula* Cushman, 1922; **10, 11**. *Bolivina* sp. 1; 12, 13. *Bolivina* sp. 2; **14–16**. *Rectuvigerina phlegeri* Le Calvez, 1959; **17–22**. *Ammonia convexa* Collins, 1978; **23–27**. *Ammonia aoteana* (Finlay, 1940); **28, 29**. *Cibicides pseudolobatulus* Perelis and Reiss, 1975; **30**. *Amphistegina* sp. 1. Scale bar is 100 μm for all magnifications and 50 μm for Figs 13.1, 13.2, 13.9 and 13.11.

This study provides the first quantitative, species-level and lagoon-wide survey of modern benthic foraminifera present in Lagos Lagoon. A total of 42 species and 25 genera of benthic foraminifera were documented (Figs 10–13). The species recorded include 10 agglutinated, 10 porcelaneous, and 22 hyaline perforate taxa.

### Spatial distribution: Salinity

Our data revealed a distinct separation of assemblages dominated by agglutinated and hyaline-perforate/porcelaneous taxa across the lagoon (Fig 2). Assemblages with predominantly high percent abundances of agglutinated taxa prevail along the northern lagoon shores, in freshwater diluted waters along the fan-like wedge created by the outflow of the Ogun River and in the eastern sector of Lagos Lagoon. Throughout the year, these areas show the lowest salinity values within Lagos Lagoon ranging from freshwater at the mouth of the Ogun River to low saline brackish water conditions (~10‰). Agglutinated foraminifera were recorded to constitute ~90% of the assemblages at all sites in the eastern sector and in front of the Ogun River mouth and more than 67% along the central northern lagoon shores. Assemblages dominated by hyaline-perforate taxa prevail around the harbor and in Commodore entrance channel, in shallow waters of the densely populated southwestern lagoon area, and along the central southern shores of Lagos Lagoon. The latter areas also contain minor fractions of porcelaneous taxa. All sites that are dominated by hyaline-perforate taxa are marked by higher salinity values (16–30‰) and characterize the areas that are influenced by Atlantic waters entering the lagoon through the Commodore Channel, the Five Cowries Creek including portions of the central lagoon area.

The transition from characteristic agglutinated assemblages to hyaline-perforate/porcelaneous biotas appears to occur relatively abruptly, even though transitional zones are observable. Culver [30] and Langer and Lipps [31, 32] reported that such changes may occur within a few meters and that zonations are controlled by salinity [33, 34].

In general, marginal marine environments were previously shown to be dominated by agglutinated taxa [see 34 for review] and only a few calcareous taxa (e.g., ammoniids, elphidiids), survive under permanently low salinity conditions (e.g., [35, 36]). Typical end-members along decreasing salinity gradients are a few agglutinated foraminifera, including *Jadammina*, *Miliammina*, *Ammotium*). Habura et al. [37] argue that the combination of reduced salinity and low pH conditions disfavor calcification in foraminiferans, as they are dependent on the local carbonate concentration [38], which generally decreases in low salinity environments (see also [39]).

Foraminiferal wall structure types were found to have a marked salinity-dependent distribution throughout the lagoon. This phenomenon is observed in total assemblages (Fig 2) and also feature prominently in the antagonistic distribution patterns of the agglutinated species *Ammotium salsum* and the hyaline-perforate *Ammonia aoteana* (Fig 3A and 3C). While *Ammotium salsum* was found to be the dominant constituent in the low salinity northern and eastern sectors, *Ammonia aoteana* prevails along marine influenced southwestern and southcentral lagoon sites. The results of antagonistic distribution patterns recorded in total assemblages are in accordance with observations on living foraminifera in the respective areas [7, 8].

*Ammotium salsum* is known for its tolerance to salinity fluctuations, and a typical representative in marginal marine environments (e.g., [13, 24, 30, 34, 40–42]). Similar to our findings, Debenay [40] reported the species to be the dominant component (>90%) within the hyposaline (5–10‰) innermost parts of Ebrie Lagoon (Ivory Coast, W-Africa). Within the low saline Camaronera Lagoon (Gulf of Mexico), a lagoon that has not direct link to the ocean and is connected to the Gulf only via a small channel over the Alvarado Lagoon, Phleger and Lankford [43] documented that *Ammotium salsum* also constitutes ~90% of the total assemblages. In the hyposaline Ologe Lagoon (Nigeria) Fajemila and Langer [24] reported *Ammotium salsum* to represent up to 85% of the total assemblage.

Among the calcareous taxa recovered are two species of *Ammonia* (*Ammonia aoteana*, *Ammonia convexa*). Species of *Ammonia* are well known for their tolerance to salinity fluctuations [27, 28, 44]. They are present in in almost every shallow-water marginal marine and tidal influenced estuarine environments, under normal and brackish water conditions and in places where significant loads of freshwater river discharge mix with marine waters [28, 31, 34, 45]. Along the transect studied in the Ebrie Lagoon (Ivory Coast), Debenay [40] reported *Ammonia* to be present to salinity levels of ~10‰, but lacking at values below this threshold. Similarly, Fajemila and Langer [24] did not find *Ammonia* in the hyposaline Ologe Lagoon west of Lagos, but widely present along Atlantic coastline sites in the Gulf of Guinea [13, 23, 25].

Our finding of largely disjunct distribution patterns of agglutinated and hyaline-perforate/miliolid foraminifera are strongly supported by independent lines of evidence. This includes Q- and R- mode cluster, DCA, an, PCA analyses, showing distinct separations along the lines of different wall structures. This suggests, that the general distribution pattern and division of agglutinated and hyaline-perforate/porcelaneous foraminiferal biotas, are mainly driven by the salinity gradient.

### Abundance

The number of foraminifera per gram sediment (FN) was found to vary substantially among individual samples. The general pattern recorded revealed highest numbers of specimens (>300 g^-1^) in the eastern sector and in proximity to the fan-like outflow of the Ogun River, where gravitational settling promotes the deposition of fine-grained, organically enriched sediments and where seasonal bottom water hypoxia are known to occur (ST22-ST26; [12, 23]). FN numbers in these areas were found to be distinctly higher than in all other habitats and may reach up to 1000 individuals per gram sediment. The Lagos Lagoon areas that revealed the lowest FN values (<20 g^-1^), cover the industrial areas around the Commodore entrance channel, the Lagos Harbor and three sites along the southern shores near Lekki. A low FN number (19.5 g^-1^) also characterizes site ST6, a locality that is in the immediate vicinity of dense mangrove forest. Sample site ST6 was also found to stand out as an outlier in the Q-mode analyses, based on its distinct faunal composition. The site is situated at the mouth of the Ogudu Creek, where untreated municipal and industrial effluents from densely populated areas are discharged into the lagoon system. Its neighboring site ST7, in turn, a locality that is not under the impact of the Ogudu Creek, shows an FN value of 821 g^-1^. The FN numbers reported by Phillips et al. [8] were found to range between 0.6–45.8 g^-1^, agree with our findings from the polluted western lagoon sites, but are considerably lower than the high numbers reported here from less-polluted sites. Whether the striking FN differences of high and low FN are indeed a consequence of pollution, or a result of higher survivability or preservation rates, requires further study.

### Diversity

Species richness and Fisher α diversity index values recorded across the Lagos Lagoon were found to largely correlate with the disjunct distribution pattern of agglutinated and hyaline-perforate/porcelaneous taxa, with highest values around the Lagos Harbor and decreasing values towards the distal eastern sector and the northwestern area in front the Ogun River. Foraminiferal diversity thus traces the general Lagos Lagoon salinity pattern, with high diversity in areas that are under the influence of marine waters and low diversity in the low salinity areas (see also [8, 46]). Indicator taxa for the latter include *Ammotium salsum*, *Ammotium sp*. *1*, *Ammobaculites exiguus*, and *Miliammina fusca* (see also [23]). Species indicative for areas characterized by higher salinity (>16‰), include members of *Quinqueloculina*, *Miliolinella*, *Elphidium*, *Bolivina*, *Rectuvigerina*, *Triloculina*, *Textularia*, *Porosononion*, *Nonion*, *Neoeponides* and others (see [8, 9, 46]). Almost all of them have a calcareous test and are typical constituents of the shallow water marine fauna in the Gulf of Guinea [13, 25]. The high diversity recorded around the heavily polluted harbor area, appears to be contradictory at first glance, as impacted sites commonly display a decrease in the number of species, reduced abundances and a selection towards pollution-resistant taxa [47–50]. Due to the proximity to the Atlantic Ocean, the harbor is subjected to diurnal tidal fluctuations and is regularly flushed by marine waters entering through the main channel. The high diversity recordings around the harbor area is mainly driven by the presence of typical marine taxa (see also [9]), and promoted by favorable salinity and pH conditions. The extent to which seawater enters the lagoon depends on the season and brackish water conditions may exist up to >30 km into the lagoon and creeks. Within the lagoon, waters are rapidly diluted and foraminifera species richness is substantially reduced, ultimately resulting in low-diverse agglutinated assemblages (Fig 5; see also [6]). Low-diverse agglutinated assemblages, similar to the biotas recorded near the Ogun River, were recently recorded as far as 40km inland [24]. In 2012, Phillips et al. [8] recorded only 9 species of benthic foraminifera across the lagoon, with a 95% dominance of *Ammonia* in the total assemblages. In a more recent study, the number has increased to 20 benthic taxa [9]. Benthic foraminiferal species richness recorded here was found to exceed 40 and thus more than doubles previous species counts.

Anthropogenic Influences Superimposed on the salinity-driven distributional differences between agglutinated and hyaline-perforate/porcelaneous assemblages are a multitude of stressors related to increasing anthropogenic influences. The City of Lagos has experienced tremendous growth over the past 50 years, reaching ~20 million from just 1.4 million in 1970. The exponential population growth goes hand in hand with the environmental degradation of Lagos Lagoon, where present-day water pollution levels often exceed compliance levels of regulatory health standards (e.g., [3, 51–56]). Pollution levels of the lagoon were reported to be greatest in the Lagos Harbor area, along the densely populated western coast, and decreases towards the northern and the eastern sector [57]. The Lagos Lagoon ecosystem therefore includes areas that cover the full range from strongly impacted by human activities to those having low levels of direct impact [58]. The environmental impact includes pollution from untreated wastewater discharge, hydrocarbon pollutants and petroleum exploitation wastes, chemical contaminants, widespread and unregulated practice of coastal solid waste dumping, uncontrolled chemicals used by local fisherman, ineffective sewerage systems, industrial discharges, heavy metal pollution, wood residue leachates, and sand dredging activities [1, 56, 59–62] and pose a serious threat to biodiversity and the aquatic ecosystem.

### Influence of pollution

Studies on the concentration of heavy metal sediment contamination (Cd, Co, Cr, Cu, Ni, Pb, Zn), indicated i.) highest values in close proximity to anthropogenic activities and near point sources from industrial effluents and domestic sewage, ii.) spatial variation, and iii.) concentrations via bioaccumulation that exceed toxicity levels with considerable risks to aquatic systems and biotas [11, 55, 59, 63, 64].

The spatial distribution of individual species recorded along the polluted western coast of Lagos Lagoon revealed species abundance patterns that highlight selected taxa as potential bioindicators of stressed environments (Fig 3). *Ammotium salsum*, a typical representative of brackish water lagoons, was found to dominate the northwestern and eastern sector of the lagoon, where relatively lower degrees of pollution and more pristine waters are found. The species is significantly less abundant along the polluted western coast, and largely absent from the Lagos Harbor, the Commodore Channel and in shallow waters north of Lekki.

Among the sample stations situated along the polluted western coastline is site ST6, a site that is located in front of the Ogudu Creek, south of the northern mangrove area. The creek discharges municipal and industrial effluents, wastewater from the cottage industry and is used a dumpsite for solid wastes [3, 51, 65]. Q-mode cluster analysis has identified site ST6 as a distinct outlier and contains only *Trochammina* sp. 1 and two species of *Ammonia*. Besides having extremely low diversity, the site is characterized by particularly low foraminiferal abundances (FN). Studies by Ejimadu et al. [66] revealed very high levels of suspended solids (4170 ppm) that possibly result from upstream artisanal sand mining activities. Moreover, the levels of sulphate, phosphate and the concentrations of heavy metals (Fe, Cu, Pb and Zn) were found to exceed the standards of the National Environmental Standards and Regulations Enforcement Agency (NESREA). ST6 is strikingly different from its neighboring site ST7, a locality that is also near the same mangrove area but not under the impact of the Ogudu Creek. Site ST7 shows a 40-fold higher FN value and contains 8 species.

*Ammonia aotena* was found to be the dominant taxon along the highly polluted area north of Lagos Harbor (ST4), a finding that is concordance with the presence of living individuals from this area [7, 8]. The area is impacted by highest concentrations of both carcinogenic polycyclic aromatic hydrocarbons (PAH), heavy metal pollutants, trichloroethylene and characterized by higher sediment oxygen demand (SOD) and total organic matter (TOM) levels in proximity to the heavily industrialized port area near Apapa [4, 58, 64, 67]. Studies on the microbial assemblages indicated that the bacterial communities around this area differ substantially from non-polluted sites in the lagoon [58] and revealed lower diversity and evenness in the microbial communities. *Ammonia aoteana* and *A*. *convexa* often co-occur together along the industrialized western (sampling sites 1, 2, 3 and 6) and southern coast (sampling sites 15, 18 and 21) of the lagoon. Even when they occur separately, these species are most prominent in the western and southern parts of the lagoon (Fig 2 - *A*. *aoteana* at sampling sites 17 and 19; *A*. *convexa* at sampling site 8).

Species of *Ammonia* were widely reported to be among the most pollution-tolerant benthic taxa [47, 48, 50, 68]. The resilience includes tolerance against industrial and municipal sewage outlets, chemical and thermal effluents, fertilizer byproducts, oil discharges, mining effluents, land reclamation activities, and pollution site outfalls discharging heavy metals. Depending on the type and degree of pollution, the stress-related response of *Ammonia* was shown to vary from site to site, and often involves either an increase or decrease in abundance and various forms of test deformation [47, 48, 50]. The resilience of some species of *Ammonia* is indicative for higher rates of survivability under conditions of environmental perturbation, and the taxa were regarded as endmembers under extreme conditions and as potential bioindicators of pollution [47].

Other known bioindicators of pollution [47] were also recorded in Lagos Lagoon (Table 3; [7–9]). This includes species of *Elphidium*, *Nonion*, *Trochammina*, *Ammobaculites* and a few bolivinid taxa. Most of them were found to be rare but their occurrence is largely restricted to the polluted western portion of the Lagoon (Fig 2) and most of them are absent from the less-polluted eastern sector and along the northwestern outflow of the Ogun River. Species of these genera were found to tolerate discharges from both industrial and domestic effluents, including drainage of heavy metals, toxic oil-based components, sewage, and agricultural and fertilizer pollutants (reviewed in [47] and [50]). Increased abundances of pollution-associated foraminifera around impacted sites were attributed to enhanced tolerance levels, where species profit from the reduced competitive ability over more sensitive taxa, through reduced competition or predatory pressure [47, 69, 70]. Recordings of the above-mentioned genera from the innermost harbor areas (not studied here but see [9]) deserve particular attention in future biomonitoring studies.

A general pattern that emerged from this study was that the polluted western coast sites (ST1-ST4, ST6) displayed low and very low FN numbers that range from 0.6 to 61 g^-1^. The finding is in accordance with a recent study, that revealed FN numbers ranging from 0.6 to 45.8 g^-1^ in the most polluted area harbor areas [9]. Low foraminiferal abundances were also recorded from other sites impacted by oil discharges (PAH’s) and heavy metals [68, 71–74] and commonly goes hand in hand with a decrease in species richness [48, 50]. In severely polluted but regularly flushed harbor areas, where rates of seawater exchange are high and water residence time remains short, high diversity assemblages, composed of both marine, brackish and marginal marine biotas, continue to be promoted [75]. The presence of marine taxa and comparatively high species richness recorded around the Lagos Harbor area (Table 2, Fig 5), are in agreement with this finding and attest that threshold values of pollution are limited to the extent, that allow a selected number of species to thrive under the impact of antropogenically induced perturbations. Reports of harbor samples barren of foraminifera [9], indicate, however, that source-point specific biomonitoring deserves particular attention.

### Redistribution of species

The present-day Lagos Lagoon is a highly dynamic system, where sediments are injected from fluctuating river flows, disperse and settle along prevailing current regimes, are remobilized, redeposited, and accumulate on the lagoon floor or mix with incoming marine sediments from the Atlantic around the Commodore entrance channel or through the Five Cowries Creek. In general terms, the distribution of lagoon sediments follows the energy systems, with the coarsest sediments near Ogun River mouth and around the Atlantic entrance channel and the finest sediments in the innermost reaches and eastern sector, where current velocities are low and approach zero. The dissemination and redistribution of taxa is therefore of concern for biomonitoring studies, especially when working with total assemblages. The following findings, however, support the notion that collected foraminiferal biotas represent mostly autochthonous assemblages and that large scale-redistribution effects can be excluded.

1. The transition from characteristic agglutinated assemblages to hyaline-perforate/porcelaneous biotas was found to be consistent and comparatively abrupt.
2. Numerically abundant species with restricted distribution ranges (e.g., *Miliammina fusca*, *Hanzawaia* cf. *H*. *nipponica*, *Nonion fabum*, *Quinqueloculina* species) do not show random or scattered occurrences, but remain limited to confined environments.
3. Visual inspection of the foraminiferal material revealed that test preservation within the lagoon generally ranged between well-preserved and excellent. This also includes taxa with particularly fragile tests (e.g., *Miliammina fusca*) Near the Atlantic entrance and in the Commodore entrance channel, test preservation was moderate to low, indicative reworking and horizontal transport. Previous studies have shown that the degree of test preservation in foraminfera can be used as an approximation for transport rates, where well preserved tests are indicative for the living or *in situ* fauna, whereas poorly preserved tests show allochthonous origins or reworking [76, 77].
4. Hyaline-perforate and porcelaneous taxa are largely restricted to environments that are under the influence of marine waters with salinities above ~16‰.
5. Larger symbiont bearing foraminifera were found to be extremely rare (only 4 specimens), thus excluding large-scale transport of fully marine species into the lagoon habitat through the entrance channel.
6. High abundances of agglutinated foraminifera were recorded in shallow waters off Banana Island, a man-made island developed by land reclamation involving dredging activities from around the northwestern Ikorudu area. Typical foraminiferal indicator taxa present around the northwestern city of Ikorudu (ST8), were also found at site ST11 off Banana Island (e.g., *Ammotium salsum*, *Ammobaculites exiguus*). Whether sand dredging is indeed the source for the high abundance of agglutinated assemblages off Banana Island, or if the assemblage at ST11 is autochthonous, requires further study. Other than this, we do not have evidence that sand dredging has caused large-scale distortions in the general distribution pattern of foraminiferal assemblages.

In summary, this implies that the current-, wave-, or anthropogenically induced redistribution of taxa is limited and that the benthic assemblages may preserve the original community structures and sufficient environmental information to be useful in biomonitoring studies.

## Conclusions

Lagos Lagoon is the ultimate sink for its metropolitan residential and industrial discharges and a significant repository of pollutants. Our study on the spatial distribution, species richness, structural composition and abundance of individual taxa of benthic foraminifera, leads to the following major conclusions:

1. Lagos Lagoon houses a total of 42 species of benthic foraminifera including 10 porcelaneous, 22 hyaline perforate and 10 agglutinated species. Our research constitutes the most comprehensive study on benthic foraminifera with species records that more than double previous species counts.
2. Foraminiferal assemblages recorded across the lagoon display a two-part pattern that is separated along the lines of wall structural types. Agglutinated foraminifera strongly dominate in the low saline eastern and northwestern portion the lagoon and foraminifera with a hyaline-perforate or porcelaneous test are mainly present in the marine influenced areas. The spatial separation of lagoonal biotas into two domains is supported by independent lines of evidence, including cluster, PCA and DCA analysis, and features prominently in the antagonistic distribution patterns of the two most abundant taxa (*Ammotium salsum*, *Ammonia aoteana*) and in FN recordings. The spatial separation is largely oriented along salinity contour lines, does not co-vary with pH and TDS, and appears to be largely driven by salinity.
3. Areas with high pollution along the highly populated western and southwestern coasts were found to be characterized by low FNs but comparatively higher species richness values. High diversity recordings around the polluted harbor area is mainly driven by the presence of marine taxa, indicative for the influence of Atlantic waters entering the harbor area through the main entrance channel.
4. Analysis of total assemblages shows the foraminiferal biotas to be largely autochtonous, and thus preserve the original community structures and sufficient environmental information to be useful in paleoecology.
5. The sites impacted by pollution were found to be characterized by specific assemblages and taxa, indicative for enhanced tolerance levels to multiple stressors, and provide a repertoire of bioindicators to assist in future studies on environmental perturbations.

## Supporting information

S1 Appendix Benthic foraminifera count from the sediments of the Lagos Lagoon sample stations. (DOCX)
