# Supplementary material for: Spatial distribution of benthic foraminifera in the Lagos Lagoon (Nigeria): Tracing the impact of environmental perturbations
Source: PLoS One. 2020 Dec 7;15(12):e0243481. doi: 10.1371/journal.pone.0243481 (PMC7721140; doi:10.1371/journal.pone.0243481)
Supplement: S1 Appendix — (DOCX) [file pone.0243481.s001.docx]

.

| **Species** | **ST1** | **ST2** | **ST3** | **ST4** | **ST5** | **ST6** | **ST7** | **ST8** | **ST9** | **ST10** | **ST11** | **ST14** | **ST15** | **ST16** | **ST17** | **ST18** | **ST19** | **ST20** | **ST21** | **ST22** | **ST23** | **ST24** | **ST25** | **ST26** |
| --- | --- | --- | --- | --- | --- | --- | --- | --- | --- | --- | --- | --- | --- | --- | --- | --- | --- | --- | --- | --- | --- | --- | --- | --- |
| *Ammobaculites* *exiguus* | 0 | 0 | 0 | 0 | 14 | 0 | 14 | 6 | 3 | 13 | 19 | 0 | 0 | 17 | 0 | 0 | 8 | 9 | 2 | 26 | 15 | 28 | 19 | 31 |
| *Ammonia convexa* | 1 | 6 | 0 | 24 | 4 | 2 | 1 | 4 | 3 | 8 | 1 | 0 | 9 | 7 | 0 | 7 | 4 | 3 | 21 | 4 | 5 | 8 | 14 | 20 |
| *Ammonia aoteana* | 9 | 16 | 0 | 139 | 13 | 4 | 9 | 2 | 5 | 5 | 2 | 0 | 21 | 21 | 9 | 61 | 47 | 15 | 117 | 3 | 29 | 8 | 5 | 7 |
| *Ammontium salsum* | 1 | 1 | 0 | 12 | 151 | 0 | 227 | 24 | 53 | 182 | 231 | 1 | 9 | 117 | 11 | 0 | 15 | 87 | 39 | 286 | 244 | 255 | 189 | 255 |
| *Ammotium* sp.1 | 0 | 0 | 0 | 0 | 2 | 0 | 6 | 8 | 2 | 2 | 3 | 0 | 0 | 3 | 0 | 0 | 5 | 0 | 2 | 3 | 6 | 3 | 2 | 1 |
| *Amphistegina* sp. 1 | 0 | 0 | 0 | 0 | 0 | 0 | 0 | 0 | 0 | 0 | 0 | 0 | 0 | 1 | 0 | 0 | 0 | 0 | 0 | 0 | 0 | 0 | 0 | 0 |
| *Bolivina* cf. *B*. *persiensis* | 0 | 1 | 0 | 0 | 0 | 0 | 0 | 0 | 0 | 0 | 0 | 0 | 0 | 1 | 0 | 0 | 0 | 0 | 1 | 0 | 0 | 0 | 0 | 0 |
| *Bolivina striatula* | 0 | 1 | 0 | 1 | 0 | 0 | 0 | 0 | 0 | 0 | 0 | 0 | 0 | 0 | 0 | 0 | 0 | 0 | 0 | 0 | 0 | 0 | 0 | 0 |
| *Bolivina* sp. 1 | 0 | 0 | 0 | 0 | 0 | 0 | 0 | 0 | 0 | 0 | 0 | 0 | 1 | 0 | 0 | 0 | 0 | 0 | 0 | 0 | 0 | 0 | 0 | 0 |
| *Bolivina* sp. 2 | 0 | 0 | 0 | 0 | 0 | 0 | 0 | 0 | 0 | 0 | 0 | 0 | 0 | 1 | 0 | 0 | 0 | 0 | 0 | 0 | 0 | 0 | 0 | 0 |
| *Caronia exilis* | 0 | 1 | 0 | 0 | 0 | 0 | 0 | 0 | 0 | 0 | 0 | 0 | 0 | 2 | 0 | 0 | 0 | 0 | 0 | 0 | 1 | 0 | 0 | 0 |
| *Cibicides pseudolobatulus* | 0 | 0 | 1 | 0 | 1 | 0 | 0 | 0 | 0 | 0 | 0 | 0 | 1 | 0 | 0 | 0 | 0 | 0 | 0 | 0 | 0 | 0 | 0 | 0 |
| *Cribroelphidium mirum* | 0 | 6 | 0 | 4 | 4 | 0 | 0 | 0 | 0 | 0 | 0 | 0 | 4 | 7 | 0 | 0 | 0 | 0 | 0 | 0 | 7 | 0 | 0 | 0 |
| *Edentosomina* sp. 1 | 0 | 1 | 0 | 0 | 0 | 0 | 0 | 0 | 0 | 0 | 0 | 0 | 0 | 0 | 0 | 0 | 0 | 0 | 0 | 0 | 0 | 0 | 0 | 0 |
| *Elphidium* sp. 1 | 0 | 0 | 0 | 0 | 0 | 0 | 0 | 0 | 0 | 0 | 0 | 0 | 1 | 1 | 0 | 0 | 0 | 0 | 0 | 0 | 0 | 0 | 0 | 0 |
| *Globocassidulina*? sp. 1 | 0 | 0 | 0 | 0 | 0 | 0 | 0 | 0 | 0 | 0 | 0 | 0 | 1 | 0 | 0 | 0 | 0 | 0 | 0 | 0 | 0 | 0 | 0 | 0 |
| *Hanzawaia* cf*. H. nipponica* | 1 | 6 | 1 | 1 | 0 | 0 | 0 | 0 | 0 | 0 | 0 | 0 | 1 | 6 | 0 | 0 | 2 | 4 | 1 | 0 | 0 | 0 | 0 | 0 |
| *Miliammina fusca* | 0 | 0 | 0 | 0 | 0 | 0 | 5 | 6 | 1 | 0 | 0 | 0 | 0 | 0 | 0 | 0 | 0 | 0 | 0 | 3 | 2 | 0 | 1 | 0 |
| *Miliolinella* sp.1 | 0 | 1 | 0 | 0 | 0 | 0 | 0 | 0 | 0 | 0 | 0 | 0 | 1 | 0 | 0 | 0 | 0 | 0 | 0 | 0 | 0 | 0 | 0 | 0 |
| *Neoeponides* sp. | 1 | 2 | 0 | 0 | 2 | 0 | 0 | 0 | 1 | 0 | 0 | 0 | 1 | 3 | 0 | 0 | 0 | 2 | 0 | 0 | 0 | 0 | 0 | 0 |
| *Nonion fabum* | 1 | 8 | 1 | 0 | 0 | 0 | 0 | 0 | 0 | 0 | 0 | 0 | 0 | 3 | 0 | 0 | 0 | 1 | 7 | 0 | 0 | 0 | 0 | 0 |
| *Pararotalia* *sarmientoi* | 0 | 0 | 0 | 0 | 0 | 0 | 0 | 0 | 0 | 1 | 0 | 0 | 1 | 1 | 0 | 0 | 0 | 0 | 0 | 0 | 0 | 0 | 0 | 0 |
| *Planulina* sp*. 1* | 0 | 0 | 0 | 0 | 0 | 0 | 0 | 0 | 0 | 0 | 0 | 0 | 1 | 0 | 0 | 0 | 0 | 0 | 0 | 0 | 0 | 0 | 0 | 0 |
| *Porosononion* sp. 1 | 0 | 0 | 0 | 0 | 0 | 0 | 0 | 0 | 0 | 0 | 0 | 0 | 1 | 0 | 0 | 0 | 0 | 0 | 0 | 0 | 0 | 0 | 0 | 0 |
| *Pseudotriloculina* sp. 1 | 0 | 1 | 0 | 0 | 0 | 0 | 0 | 0 | 0 | 0 | 0 | 0 | 0 | 0 | 0 | 0 | 0 | 0 | 0 | 0 | 0 | 0 | 0 | 0 |
| *Quinqueloculina* cf. *Q*. *cuvieriana* | 1 | 1 | 0 | 0 | 0 | 0 | 0 | 0 | 0 | 0 | 0 | 0 | 1 | 0 | 0 | 0 | 0 | 0 | 0 | 0 | 0 | 0 | 0 | 0 |
| *Quinqueloculina* cf. *Q*. *seminulum* | 1 | 3 | 0 | 0 | 1 | 0 | 0 | 0 | 0 | 0 | 0 | 0 | 1 | 0 | 0 | 0 | 1 | 0 | 1 | 0 | 0 | 0 | 0 | 0 |
| *Quinqueloculina debenayi* | 0 | 0 | 0 | 0 | 0 | 0 | 0 | 0 | 0 | 0 | 0 | 0 | 0 | 0 | 0 | 0 | 0 | 0 | 1 | 0 | 0 | 0 | 0 | 0 |
| *Quinqueloculina seminulum* | 0 | 2 | 0 | 0 | 1 | 0 | 0 | 0 | 0 | 0 | 0 | 0 | 1 | 3 | 0 | 0 | 0 | 4 | 1 | 0 | 0 | 0 | 0 | 0 |
| *Quinqueloculina* sp. 1 | 0 | 0 | 0 | 1 | 0 | 0 | 0 | 0 | 0 | 0 | 0 | 0 | 0 | 0 | 0 | 0 | 0 | 1 | 0 | 0 | 0 | 0 | 0 | 0 |
| *Quinqueloculina* sp. 2 | 0 | 2 | 0 | 0 | 0 | 0 | 0 | 0 | 0 | 0 | 0 | 0 | 1 | 1 | 0 | 0 | 0 | 0 | 0 | 0 | 0 | 0 | 0 | 0 |
| *Quinqueloculina* sp. 3 | 0 | 1 | 0 | 0 | 0 | 0 | 0 | 0 | 0 | 0 | 0 | 0 | 0 | 0 | 0 | 0 | 0 | 0 | 0 | 0 | 0 | 0 | 0 | 0 |
| *Quinqueloculina* sp. 4 | 1 | 2 | 0 | 0 | 1 | 0 | 0 | 0 | 0 | 0 | 0 | 0 | 0 | 1 | 0 | 0 | 0 | 0 | 1 | 0 | 0 | 0 | 0 | 0 |
| *Rectuvigerina phlegeri* | 0 | 0 | 0 | 0 | 0 | 0 | 0 | 0 | 0 | 0 | 0 | 0 | 1 | 0 | 0 | 0 | 0 | 0 | 0 | 0 | 0 | 0 | 0 | 0 |
| Rosalina cf R. *orientalis* | 0 | 1 | 0 | 0 | 0 | 0 | 0 | 0 | 0 | 0 | 0 | 0 | 0 | 1 | 0 | 0 | 0 | 0 | 0 | 0 | 0 | 0 | 0 | 0 |
| *Rotorbis*? sp. 1 | 0 | 1 | 0 | 0 | 0 | 0 | 0 | 0 | 0 | 0 | 0 | 0 | 0 | 0 | 0 | 0 | 0 | 0 | 0 | 0 | 0 | 0 | 0 | 0 |
| *Siphotextularia* sp. 1 | 1 | 1 | 0 | 0 | 0 | 0 | 0 | 0 | 0 | 0 | 0 | 0 | 0 | 0 | 0 | 0 | 0 | 0 | 0 | 0 | 0 | 0 | 0 | 0 |
| *Textularia* *candeiana* | 0 | 0 | 0 | 0 | 0 | 0 | 0 | 0 | 0 | 0 | 0 | 0 | 2 | 1 | 0 | 0 | 0 | 0 | 0 | 0 | 0 | 0 | 0 | 0 |
| *Textularia* sp. 1 | 6 | 2 | 0 | 0 | 0 | 0 | 0 | 0 | 0 | 0 | 0 | 0 | 5 | 10 | 0 | 0 | 0 | 3 | 2 | 0 | 0 | 0 | 0 | 0 |
| *Textularia* sp. 2 | 3 | 0 | 0 | 0 | 0 | 0 | 0 | 0 | 0 | 0 | 0 | 0 | 0 | 1 | 0 | 0 | 0 | 0 | 0 | 0 | 0 | 0 | 0 | 0 |
| *Trilocularena patensis* | 0 | 0 | 0 | 0 | 0 | 0 | 0 | 0 | 0 | 0 | 0 | 0 | 0 | 0 | 0 | 0 | 0 | 0 | 0 | 2 | 0 | 0 | 0 | 0 |
| *Triloculina* sp. 1 | 0 | 1 | 0 | 0 | 0 | 0 | 0 | 0 | 0 | 0 | 0 | 0 | 0 | 0 | 0 | 0 | 0 | 0 | 0 | 0 | 0 | 0 | 0 | 0 |
| *Trochammina* sp. 1 | 0 | 0 | 0 | 7 | 43 | 9 | 1 | 0 | 0 | 47 | 46 | 1 | 0 | 4 | 0 | 0 | 2 | 0 | 0 | 1 | 26 | 47 | 32 | 27 |

S1 Appendix. Benthic Foraminifera count data obtained from Lagos Lagoon sediment samples
